# Comparative analysis of expressed sequence tags (ESTs) between drought-tolerant and -susceptible genotypes of chickpea under terminal drought stress

**DOI:** 10.1186/1471-2229-11-70

**Published:** 2011-04-22

**Authors:** Amit A Deokar, Vishwajith Kondawar, Pradeep K Jain, S Mohan Karuppayil, N L Raju, Vincent Vadez, Rajeev K Varshney, R Srinivasan

**Affiliations:** 1 National Research Center on Plant Biotechnology, IARI Campus, New Delhi 110012, India; 2 School of Life Sciences, S.R.T.M. University, "Dnyanteerth", Vishnupuri, Nanded - 431 606, India; 3 International Crops Research Institute for the Semi-Arid Tropics (ICRISAT), Patancheru, Greater Hyderabad 502 324, AP, India; 4 Genomics towards Gene Discovery Sub Programme, Generation Challenge Programme (GCP) c/o CIMMYT, Int. Apartado Postal 6-641, 06600, Mexico, DF Mexico

## Abstract

**Background:**

Chickpea (*Cicer arietinum *L.) is an important grain-legume crop that is mainly grown in rainfed areas, where terminal drought is a major constraint to its productivity. We generated expressed sequence tags (ESTs) by suppression subtraction hybridization (SSH) to identify differentially expressed genes in drought-tolerant and -susceptible genotypes in chickpea.

**Results:**

EST libraries were generated by SSH from root and shoot tissues of IC4958 (drought tolerant) and ICC 1882 (drought resistant) exposed to terminal drought conditions by the dry down method. SSH libraries were also constructed by using 2 sets of bulks prepared from the RNA of root tissues from selected recombinant inbred lines (RILs) (10 each) for the extreme high and low root biomass phenotype. A total of 3062 unigenes (638 contigs and 2424 singletons), 51.4% of which were novel in chickpea, were derived by cluster assembly and sequence alignment of 5949 ESTs. Only 2185 (71%) unigenes showed significant BLASTX similarity (<1E-06) in the NCBI non-redundant (nr) database. Gene ontology functional classification terms (BLASTX results and GO term), were retrieved for 2006 (92.0%) sequences, and 656 sequences were further annotated with 812 Enzyme Commission (EC) codes and were mapped to 108 different KEGG pathways. In addition, expression status of 830 unigenes in response to terminal drought stress was evaluated using macro-array (dot blots). The expression of few selected genes was validated by northern blotting and quantitative real-time PCR assay.

**Conclusion:**

Our study compares not only genes that are up- and down-regulated in a drought-tolerant genotype under terminal drought stress and a drought susceptible genotype but also between the bulks of the selected RILs exhibiting extreme phenotypes. More than 50% of the genes identified have been shown to be associated with drought stress in chickpea for the first time. This study not only serves as resource for marker discovery, but can provide a better insight into the selection of candidate genes (both up- and downregulated) associated with drought tolerance. These results can be used to identify suitable targets for manipulating the drought-tolerance trait in chickpea.

## Background

Chickpea (*Cicer arietinum *L.), the fourth most important grain-legume crop, is grown in more than 45 countries, mostly in arid and semiarid zones. Approximately 90% of the crop is grown under rainfed conditions, wherein yield is significantly affected by abiotic stresses such as drought, heat, and cold [1-3]. Drought-related yield losses can occur in 40%-60% of the total chickpea production [4]. Terminal drought, which occurs at the pod filling and seed-developing stage of the crop and increases in severity at the end of the season, is a major constraint to chickpea production [1,5,6]. The identification of differentially expressed genes between 2 genotypes differing in drought tolerance and a set of their progenies can therefore be an important indicator of drought-associated genes in chickpea.

Functional genomics approaches have been used in recent years to understand the stress-responsive mechanism in plants. Candidate genes involved in drought tolerance mechanisms have been identified, characterized, and assessed for their comparative transcriptional activity by using whole-genome sequencing or expressed sequence tag (EST) libraries. Several functional genomics studies have been performed in chickpea to identify the abiotic stress-responsive transcripts by approaches such as suppression subtractive hybridization (SSH), Super serial analysis of gene expression (SuperSAGE), microarray, and EST sequencing [7-9]. Additional file 1 summarizes results of previous studies on identifying ESTs associated with drought stress in chickpea.

SSH has been widely used to compare patterns of gene expression in tissues under different conditions. However, it has not yet been used to identify differentially expressed transcripts (both up- and downregulated) in chickpea in response to drought stress at the flowering stage of plants. In all earlier studies, except the one by Varshney et al. [9], water stress was imposed by either completely withdrawing water or allowing uprooted young seedlings to wilt at room temperature. However, under field conditions, water stress progresses gradually and a similar type of stress is simulated in the laboratory by the "dry down experiment," which allows comparison of different genotypes and their response toward drought [10]. Moreover, stress response of a plant at the seedling stage can be very different from that at the reproductive stage, the latter being an important and yield-determining stage in chickpea.

In the present study, we constructed several reciprocal SSH libraries by using drought-tolerant and -susceptible genotypes as well as extreme recombinant inbred lines (RILs) for the high root biomass (HRB) and low root biomass (LRB) under terminal drought stress. This approach differs from that used in earlier studies in the following aspects: (1) use of 2 chickpea genotypes differing in their drought-tolerance capacity and their RIL progenies; (2) drought stress imposed at the flowering stage in a gradual manner by the dry down method; (3) plant samples analyzed when each plant experienced the same amount of stress, as judged by their transpiration ratio; and (4) reciprocal subtraction of transcripts by using control and stress conditions as well as susceptible and tolerant genotypes to enable a good comparison and identify both up- and downregulated genes. Thus, the EST set we used is novel and represents genes that are up and downregulated in response to terminal drought stress, and can thereby help several genes that have not been shown to be previously associated with drought stress in chickpea. The differentially expressed ESTs were analyzed using macro-array, northern blotting, and quantitative PCR.

## Methods

### Plant Material

The drought-tolerant characteristics of chickpea line ICC 4958 and drought-susceptible characteristics of ICC 1882 have been attributed to their large and prolific and small root system, respectively. An RIL mapping population (264 RILs) of ICC 4958 (large root) and ICC 1882 (small root) has been developed and phenotyped at the International Crops Research Institute for the Semi-Arid Tropics (ICRISAT), Patancheru (17° 30' N; 78° 16' E; altitude 549 m). The root phenotyping experiment was conducted in PVC cylinders with 18 cm diameter and 120 cm height, filled with soil-sand mixture in open field conditions. Plants were sampled at 35 days after sowing and different measurements were recorded as described by Kashiwagi et al. [11]. Ten RILs for extreme phenotype of high root biomass and low root biomass were selected on the basis of phenotypic evolution [Varshney et al. unpublished] to prepare bulk cDNA SSH libraries.

### Stress treatment

#### Dry down procedure

Dry down, a gradual and progressive water deficit stress, was given to plants [10]. Experiments were conducted in triplicate in a glass house receiving natural solar radiation, with air temperature regulated between 23°C and 28°C (night/day). Seeds of ICC 4958, ICC 1882, 10 RILs each for HRB and LRB, were sown in plastic pots of 8-in. diameter. Water stress (WS) treatment was initiated 35 days after the emergence of plants. All pots were saturated with water and left overnight to drain excess water. Next day, the surfaces of pots were covered with plastic beads to prevent water loss through the soil surface. Weight (in g) of individual pots was recorded daily in the morning at approximately 10.30 h. Daily loss of water through transpiration was calculated as the difference in pot weight on the current day from that on the previous day. Control plants were maintained at approximately 80% field capacity by daily compensation of water loss due to transpiration. To expose WS plants to a progressive water deficit, they were allowed to lose a maximum of 80 g of water per day; any additional loss was compensated by adding water to the pots. The transpiration of each plant was then calculated as the difference in its weight on successive days, plus water added on the previous day. Transpiration data were analyzed as described previously [10]. Well watered (WW) pots were maintained at a normalized transpiration ratio (NTR) value of 1 and WS treatment was continued until the ratio of the transpiration of the stressed plant to the average transpiration of WW plants reached ≤0.1, that is, when the transpiration of WS plants was <1% of the WW plants, a stage defined as the endpoint for the water deficit treatment [10]. WS plants reached this stage in 10 to13 day of initiation of stress treatment. At this stage, shoot and the root tissues from WW and WS plants were separately harvested, frozen in liquid nitrogen, and stored at -80°C for RNA extraction.

### RNA and mRNA isolation

Total RNA was isolated by using the Trizol reagent (Invitrogen, Carlsbad, CA), and mRNA was further isolated by using the PolyATract mRNA Isolation System (Promega, Madison, WI). To construct bulk libraries, equal amounts of total RNA (100 μg from each RIL) isolated from 10 RILs of extreme HRB and 10 RILs of extreme LRB were pooled separately and used for mRNA isolation.

### Suppression Subtractive Hybridization (SSH)

**Figure 1:**
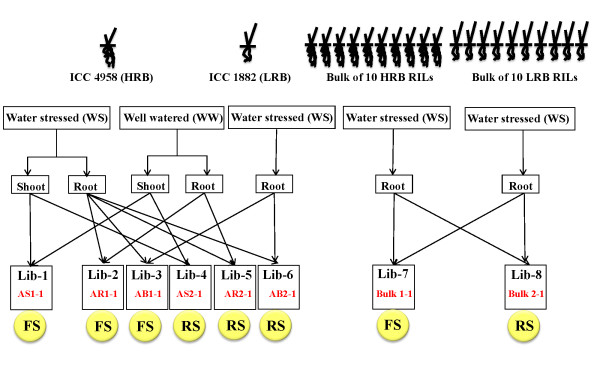
**Schematic details about the SSH libraries**. Two chickpea genotype (ICC 4958- HRB, drought resistant and ICC 1882-LRB, drought susceptible cultivar) and 10 extreme recombined inbreed lines each of HRB and LRB phenotype derived from ICC 4958 × ICC 1882 mapping population were used for construction of eight cDNA SSH libraries. Both forward (FS) and reverse subtractions (RS) were generated using reciprocal samples.

To isolate genotype and tissue-specific transcripts related to drought, 3 subtraction strategies were employed (Figure 1). In the first strategy, forward subtraction was carried out by subtracting the cDNA of WW ICC 4958 root tissue from that of the WS ICC 4958 root tissue to isolate differentially upregulated genes in roots under drought stress. Reverse subtraction was performed to isolate downregulated genes under drought stress. Similarly, forward and reverse subtractive libraries were made from the shoot tissue. In the second strategy, reciprocal subtraction of cDNA from root tissue of ICC 4958 and ICC 1882, both receiving WS treatment, was performed to isolate differentially expressed genes in the genotypes. In the third approach, cDNA from 10 RILs, each showing extreme phenotype for HRB and LRB for reciprocal subtraction, was used to isolate drought-associated differentially expressed genes in RILs exhibiting extreme root biomass phenotype.

Subtractive libraries were constructed by using the Clontech PCR-Select™ cDNA subtraction kit (Clontech, Palo Alto, CA), starting with 2 μg of mRNA from tester and drivers samples. Table 1 lists the testers and drivers used to construct 8 different SSH libraries. Forward and reverse subtraction was performed according to manufacturer's instructions to identify the transcript enriched in one sample relative to the other. Subtracted cDNAs were purified by the MinElute PCR purification kit (Qiagen, Valencia, CA) and ligated into a pGEM-T easy vector (Promega). Ligated plasmid DNAs were used for transformation into competent *E. coli *strain DH5α. Positive clones were selected on an Ampicillin/IPTG/X-Gal LB plate. Plasmid DNA from positive clones were isolated by using REAL 96 plasmid isolation kit (Qiagen), and purified DNA was used for single-pass Sanger sequencing by using T7/SP6/M13F universal sequencing primers.

**Table 1:** Summary of drought responsive SSH libraries and ESTs.

**Name of Library**	**Tester (condition/genotype/tissue)**	**Driver (Condition/genotype/tissue)**	**No of clones**	**Total no of ESTs**	**HQS**	**Type of transcripts clones**
AS1-1	WS/ICC 4958/Shoot	WW/ICC 4958/Shoot	960	807	753	Up regulated in shoot tissue under drought stress
AS2-1	WW/ICC 4958/Shoot	WS/ICC 4958/Shoot	960	877	821	Down regulated in shoot tissue under drought stress
AR1-1	WS/ICC 4958/Root	WW/ICC 4958/Root	1440	1424	1281	Up regulated in root tissue under drought stress
AR2-1	WW/ICC 4958/Root	WS/ICC 4958/Root	960	940	799	Down regulated in root tissue under drought stress
AB1-1	WS/ICC 4958/Root	WS/ICC 1882/Root	576	576	503	Up regulated in roots of resistant genotype (ICC 4958) under drought stress
AB2-1	WS/ICC 1882/Root	WS/ICC 4958/Root	576	576	529	Down regulated in roots of resistant genotype (ICC 4958) under drought stress
Bulk1-1	WS/Bulk HRB/Root	WS/Bulk LRB/Root	480	423	400	Up regulated in roots of extremes bulks of RILs of HRB under drought stress
Bulk2-1	WS/Bulk LRB/Root	WS/Bulk HRB/Root	480	429	408	Down regulated in roots of extremes bulks of RILs of HRB under drought stress
**Total ESTs**			**6432**	**6053**	**5494**	
**Total unigenes**					**3062**	

High root biomass genotype (HRB) ICC 4958 and low root biomass genotypes (LRB) ICC 1882 are tagged with "A" and "B" for library description respectively. HQS: High quality sequences, 1-1: Forward subtraction, 2-2: Reverse subtraction, WW: Well watered, WS: Water stressed plants.

### Sequence processing

All sequences were checked for quality and then analyzed by Seqman™ II 5.08 (DNASTAR, Inc.. Lasergene Gene Corporation, Ann Arbor, MI) to detect and remove pGEMT-Easy vector sequences. A Perl script EST trimmer [12] was used to trim adaptors, poly A/T ends. EST sequences which were less than 100 bp long were removed. Manual sequence processing was also performed to confirm results. ESTs from individual libraries were assembled into contigs, using default parameters of CAP3 [13]. Incorporation of ESTs into a contig required at least 95% sequence identity and a minimum 40-bp overlap. ESTs from all 8 libraries also underwent CAP3 analysis to produce a differentially expressed unigene dataset.

### Sequence annotation

The NCBI BLAST program [14] version 2.2.6 was used to perform BLASTN and BLASTX similarity searches. BLASTN analysis was performed to determine sequence homology at the nucleotide level of this unigenes set with EST databases of *Medicago truncatula, Glycine max, Lotus japonicus, and Phaseous vulgaris *and also with ESTs of model plant species such as *Arabidopsis thaliana, Oryza sativa*, and *Populus alba *downloaded from NCBI. The cutoff expectation (*E*)-value threshold for BLASTN searches was ≤1e-5. BLASTX was performed against NCBI non-redundant (nr) database using Blast2GO with an *E-*value cutoff of <1e-06.

### Functional categorization and GO enrichment analysis

Functional annotation was performed by using Blast2GO (version 2.2.3) [15], following the standard procedure of BLASTX for unigenes dataset (parameters: nr database, high scoring segment pair (HSP) cutoff length 33, report 20 hits, maximum *E-value *1.0E-3), followed by mapping and annotation (parameters: *E-value *hit filter 1.0E-6, annotation cutoff 55, GO weight 5, HSP-hit coverage cutoff 20). GO terms were summarized according to their molecular functions, biologic processes, and cellular components. Enzyme mapping of annotated sequences was performed by using direct GO to Enzyme mapping and used to query the Kyoto Encyclopaedia of Genes and Genomes (KEGG) to define the KEGG orthologs (KOs). These KOs were then plotted into the whole metabolic atlas by using the KEGG mapping tool [16].

GO enrichment analysis was performed by using the Fisher exact test, as implemented in the GOSSIP module [17] integrated in Blast2GO package. For GO enrichment analysis, all GO terms with a cut-off threshold of pFDR(p) ≤ 0.05 were considered differentially enriched between 2 set of EST libraries. To study the genotype-specific response for ICC 4958 and ICC 1882 under drought stress, GO enrichment analysis was performed between ESTs developed from the SSH libraries AB1-1 and AB2-1, which were constructed to identify transcripts induced in response to drought in the tolerant genotype ICC 4958 and the susceptible genotype ICC 1882, respectively.

### Macroarray and Northern Hybridization

To screen the differentially expressed ESTs identified in present work, two different macroarray experiments were conducted. In the first experiment, a nylon macroarray in 96-well format, using unigenes from AS1-1 and AS2-1 libraries, was constructed and total RNA from WW and WS plants of ICC 4958 were used to evaluate the differentially expressed unigenes under water stressed condition. Where as in second experiment, a nylon macroarray in a 96-well format, using unigenes from AB1-1 and AB2-1 libraries, was constructed and total RNA from water-stressed ICC 4958 and ICC 1882 were used to evaluate the genotype-specific response under water stress condition.

Equal amounts of purified PCR amplified products (100 ng) was spotted onto nylon membranes (Amersham Pharmacia Biotech, Uppsala, Sweden), using the dot-blot apparatus in 96 formats. Each blot was prepared in duplicate. PCR-amplified products of actin cDNA (GenBank: EU529707) as a housekeeping gene for normalization of the signals between the blots and neomycin phosphotransferase (NPTII) as a negative control for signal background correction were spotted on the membrane and cross-linked using UV. RNA samples were labeled during first-strand cDNA synthesis. Total RNA (5 μg ) was reverse transcribed, using SuperScript III RT enzyme (Superscript II, Life Technologies, Grand Islands, NY) in the presence of α-[^32^P] dCTP and used as probes. The nylon membrane were prehybridized with formamide hybridization buffer for 42°C for 6 h, the denatured probe was added, and hybridized for 24 h. Washed membranes were exposed to X-ray film (BIOMAX MR Film, Kodak) and developed after 7 days of incubation at -80°C. The image of the developed film was acquired by SYNGENE-G-Box gel documentation and analysis system (Syngene, Synoptics Ltd, Cambridge, UK) and signal intensity of each spot was calculated by the Gene tool software. Transcript levels for each unigenes were calculated as the average intensity from triplicate experiments. The intensity of each spot was normalized with respect to the intensity of actin gene. Change in level of expression was expressed as the expression ratio of normalized signal intensities of respective unigenes in control versus treatments. On the basis of macroarray results, genes exhibiting significant induction were validated by Northern blotting.

For northern blotting total RNA (20 μg) from WW and WS plants was separated by electrophoresis on a 1.2% FA agarose gel and transferred to an Immobilon™-Ny+ membrane (Millipore, USA) following the method of Sambrook et al. [18]. PCR-amplified individual cDNA fragments (amplified with M13 forward and reverse universal sequencing primers) were purified from the agarose gel and used as probes. cDNA-amplified actin (EU529707) was the housekeeping gene control. Probes were labeled with α32P-dCTP, using the DecaLabel™ DNA labeling kit (Fermentas Life Sciences). Northern blots were scanned using a PharosFx Plus PhosphorImager (Biorad).

### Quantitative real-time RT PCR

PCR primers for quantitative real-time PCR were designed with the parameters of optimum primer GC content of 50%, primer *T*_m _> 55-65°C, primer length 18-30 nucleotides, and an expected amplicon size of 80-200 bp (see additional file 2 for primer sequences). SYBR green qPCR was performed in 96-well plates, using the Stratagene Mx3000P system and SYBR FAST qPCR Master Mix (2x) Universal (KAPA Biosystems). All qPCR reactions were run in triplicates with a no-template control to check for contaminations. PCR was conducted under the following conditions: 3 min at 95°C (enzyme activation), 40 cycles each of 3 sec at 95°C (denaturation) and 30 s at 60°C (anneal/extend). Finally, a melting curve analysis was performed from 65° to 95°C in increments of 0.5°C, each lasting 5 s, to confirm the presence of a single product and absence of primer-dimers. Two internal controls GAPDH (glyceraldehyde-3-phosphate dehydrogenase, AJ010224) and HSP90 (GR406804) were used to normalize the variations in cDNA samples [19]. Fold changes were calculated by the 2^-δδCt ^method [20].

## Results and discussion

### Water stress treatment

A graph of NTR values of ICC 4958, ICC 1882, and 20 RILs during the stress treatment indicates that all parental lines and RILs experienced same degree of stress (Additional file 3). The dry down procedure to impose water stress in pot experiments has been successfully employed in various plant systems, including chickpea [21-25].

Considering that terminal drought is a major constraint in achieving optimal crop yields in chickpea, all experiments were conducted at the flowering stage to identify molecular responses of chickpea under water stress. In many functional genomics studies on drought response in chickpea, drought stress has been induced by withdrawing water supply or by uprooting seedlings and allowing them to wilt at room temperature [26-28]. However, the physiologic and molecular responses to these treatments are likely to be different from those experienced by the plant during natural terminal drought conditions, wherein drought stress is gradual and allows the plant to go through various stages of adaptation. Another major limitation of all these studies is the variation in the quantum of stress experienced by different plants. Depending on their genotype as well as environmental and experimental conditions, plants experience varying degrees of stress when water is withdrawn or they are allowed to wilt for a specified duration. In our study, we sampled ICC 4958 and ICC 1882 and 20 RILs at a stage when they undergo the same degree of stress, as determined by the transpiration ratio.

### cDNA SSH libraries

A total of 6432 clones were generated from the 8 SSH libraries, of which 6053 ESTs were sequenced. After a quality check, 5494 high-quality ESTs were obtained (Table 1). Four SSH libraries were constructed from resistance parent ICC 4958. In total, 2034 upregulated and 1620 downregulated ESTs were identified: 753 upregulated ESTs from library AS1-1 (shoot tissue) and 1281 from AR1-1 (root tissue), and 821 downregulated ESTs from AS2-1 (shoot tissue) and 799 from AR2-1 (root tissue). In addition, 2 reciprocal libraries were constructed using root tissues of ICC 4958 and ICC 1882: there were 503 upregulated ESTs from AB1-1 in ICC 4958 and 529 uprgulated ESTs from AB2-1 in ICC 1882. Furthermore, 400 ESTs were generated from library Bulk1-1 (constructed from the bulk of 10 extreme RILs for HRB) and 408 from library Bulk2-1 (constructed from 10 extreme RILs for LRB).

In chickpea, root growth, osmotic adjustment, and stem reserve utilization are associated with drought tolerance. Root traits such as biomass, length, density, and depth have been proposed as drought-avoidance traits under terminal drought conditions [29,30]. Roots are considered a primary site for stress signal perception, where a signaling mechanism cascade initiates gene expression in response to drought stress. These transcriptional changes can result in successful adaptations, protecting plants against environmental stress [31]. The differentially expressed ESTs identified in our study provide a list of gene regulated in response to terminal drought stress in root tissue of chickpea.

The SSH strategy can be used as an alternative and complementary transcript profiling tool to the GeneChip microarrays, especially to identify novel genes and transcripts present in low abundance [32]. Thus, the SSH technology will have more utility in a system where genome sequence information and microarray chip are not available for transcript profiling.

In 2001, 47 ESTs up- or downregulated by water stress were first identified in chickpea [33]. cDNA libraries from a drought-responsive genotype in chickpea were constructed and differentially expressed ESTs were identified using *in silico *approach [9,34]. SSH libraries have been constructed from chickpea seedling after dehydration stress [27,35] and between root tissue of 2 chickpea cultivars [36]. Transcriptome analysis by using SuperSAGE and high-throughput 454 sequencing has generated 17,493 unique 26-bp tags (SAGE UniTags) from roots of the drought-tolerant chickpea variety ICC 588 [7]. However, absence of a reference sequence for chickpea and the short read length of sequences (26-bp) limit the utility of this approach.

### EST assembly

A total 5494 high-quality sequences (average length 505 bp) were generated after removing short and low-quality sequences. A total of 3062 unigenes (638 contigs and 2424 singletons) were derived from cluster assembly and sequence alignment; each contig had 2-113 ESTs with an average length of the 527 bp. The majority of contigs (84.9%) contained 5 or fewer ESTs, whereas only 2.97% contigs were made from 20 or more ESTs (Additional file 4), indicating a high degree of normalization and subtraction efficiency. All EST sequences have been deposited in the dbEST division of GenBank (HO062174-HO068058). The unigene (UG) set developed in this study is henceforth referred to as UG-TDS (unigenes responsive to terminal drought stress). CAP3 assembly analysis of our datasets with all chickpea EST sequences (34,587) deposited in NCBI dbESTs identified 1576 unigenes (51.4% of total unigenes) as singlets and are new entries to the chickpea database.

**Figure 2:**
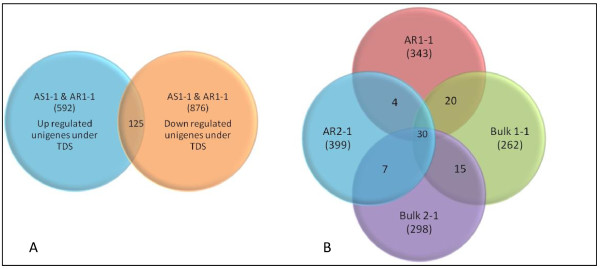
**Venn diagram representing comparison of ESTs from different SSH libraries**: (A) Cap3 assembly of four SSH libraries AS1-1(forward subtracted library from the shoots of drought tolerant genotype, ICC 4958), AR1-1(forward subtracted library from the roots of drought tolerant genotype, ICC 4958), containing up- regulated transcripts and AS2-1(reverse subtracted library from the shoots of drought tolerant genotype, ICC 4958), AR2-1(reverse subtracted library from the roots of drought tolerant genotype, ICC 4958) containing down regulated transcripts under TDS, reveals a set of 592 and 876unigenes specific to up- regulated and, down regulated libraries respectively. A set of 125 unigenes were common in both group. (B) ESTs obtained from bulk of RILs libraries Bulk 1-1(forward subtracted library from the roots of HRB and LRB) and Bulk 2-1(reverse subtracted library from the roots of HRB and LRB) and individual parental libraries, AB1-1 (forward subtracted library from the roots of ICC 4958 and ICC 1882)and AB2-1(reverse subtracted library from the roots of ICC 4958 and ICC 1882 ) reveals 343, 399, 262 and 298 unigenes specific to AB1-1, AB2-1, Bulk 1-1 and Bulk2-1 libraries, respectively.

ESTs from forward and reverse libraries were aligned to identify unique ESTs, which were up- or downregulated (*in silico *subtraction). There were 592 unigenes specific to forward-subtracted libraries and 876 unigenes to reverse-subtracted libraries. Although 125 assemblies contained ESTs from both forward and reverse libraries, this indicates very low level of redundancy between both libraries (Figure 2). ESTs identified in bulk libraries and from individual parent libraries were also aligned using CAP3 assembly, assuming that the high number of ESTs from the HRB-contributing parent ICC 4958 and bulks of RILs of the extreme HRB phenotype would form a cluster. Surprisingly, only 20 ESTs were common between ICC 4958 ESTs and bulks of RILs exhibiting HRB. Similarly, only 7 ESTs were common for ICC 1882-specific transcripts (the LRB-contributing parent in the mapping population) and the transcripts from bulks of RILs exhibiting extremes of LRB phenotype.

To determine the efficiency of normalization and subtraction of SSH libraries, we compared our ESTs with those generated by using non-normalized cDNA libraries. We have previously reported more than 20,000 chickpea root ESTs in response to drought and salt stress in ICC 4958 by using the same procedure to obtain tissue samples for constructing the libraries [9]. CAP3 assembly and clustering analysis of ESTs identified 126 contigs with 1 EST from our SSH libraries and more than 5 ESTs from non-normalized libraries. Some ESTs such as HO063066 (pathogenesis-related protein), HO063205 (plasma membrane intrinsic protein), and HO067852 (Type 1 metallothionein), had single representations in SSH libraries, whereas more than 60 clones were present in non-normalized cDNA libraries. These results support the utility and efficacy of our SSH approach to reduce the redundancy and identify specific transcripts with small-scale sequencing. Dataset analysis with all chickpea EST sequences (34,587) deposited in NCBI dbESTs identified 1576 new unigenes (51.4% of the total unigenes).

### Nucleotide-level diversity analysis

**Figure 3:**
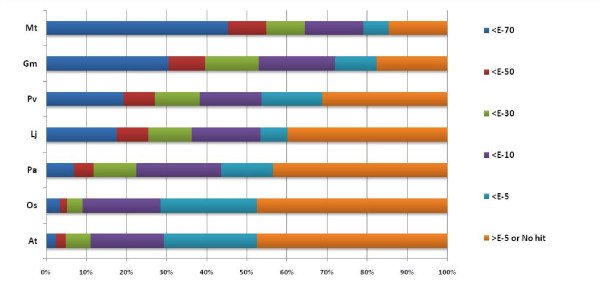
**Distribution of conservation between chickpea (*Cicer arietinum*) UG-TDS and the EST datasets of Mt (*Medicago Truncatula*), Gm (*Glycine max*), Pv (*Phaseolus vulgaris*), Lj (*Lotus japonicus*), Pa (*Populus alba*), Os (*Oryza Sativa*) and At (*Arabidopsis thaliana*)**. Unigenes were grouped according to similarity levels determined by nucleotide similarity search BLASTN E-value.

BLASTN analysis of UG-TDS revealed significant identity with *Medicago *(79.0%), followed by *Glycine max *(72.0%)*, Phaseolus *(53.7%), *Lotus *(53.4%)*, Populus *(43.6%)*, Arabidopsis *(29.4%), and *Oryza sativa *(28.5%) ESTs (Figure 3; additional file 5). Analysis of sequence similarity of chickpea UG-TDS with other legume species revealed that 2614 (85%) unigenes had significant similarity to ESTs of at least one of the analyzed legume species, with highest similarity of chickpea unigenes with *Medicago*, which is closely related to chickpea in the phylogenetic tree [37]. As expected, the 4 leguminous species showed the highest levels of similarity. The low level of sequence similarity for *L. japonicus *may be because of its EST collection is smaller (1,83,153) than those of other species such as soybean (8,80,561) and *Medicago *(1,58,131). The low nucleotide similarity observed between chickpea and other plant species does not necessarily represent phylogenetic relationships, but could depend on the coverage of EST sequences. A significant percentage of unigenes (14.6-47.5%) showing weak or no similarity (*E-value *>1E-05) for *Medicago*, *Glycine, Lotus*, and *Phaseolus*, indicating a considerable divergence in chickpea gene content within other leguminous species.

### Functional characterization of the chickpea unigene dataset

**Figure 4:**
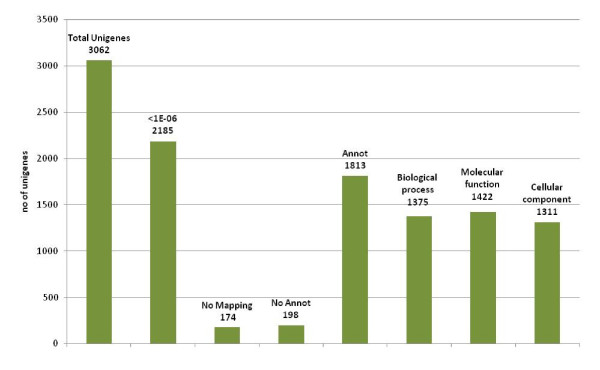
**A graphical representation of the annotation statistics of UG-TDS**: the total number of unigenes annotated as a known protein with an *E-value *threshold of e-06, total number of unigens not mapped, total number of unigenes mapped but not annotated, the total number of unigene annotated with at least one category of Gene Ontology (GO) and the number of genes annotated in each of the 3 major GO categories, biological process, molecular function and cellular component.

**Figure 5:**
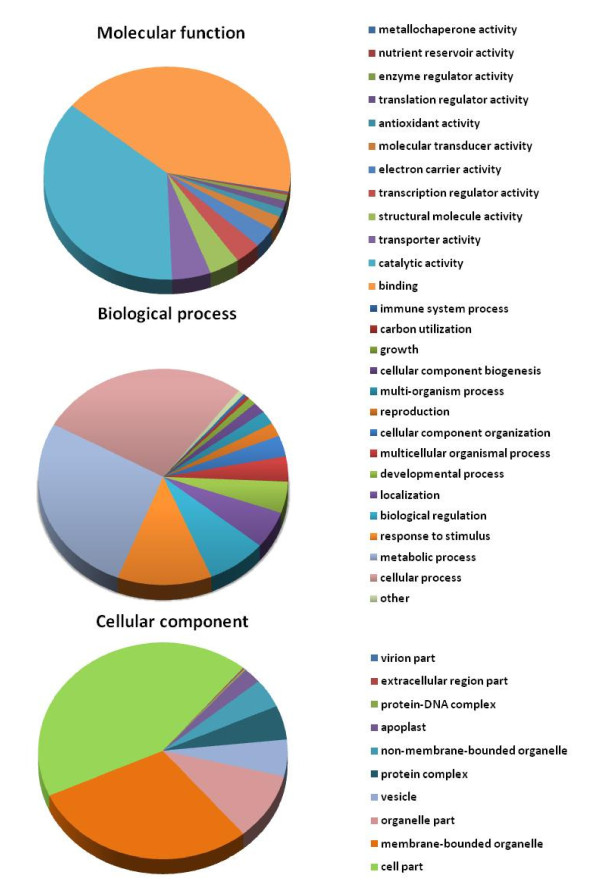
**Summary of the Gene Ontology annotation as assigned by BLAST2GO**: Gene Ontology classification of chickpea UG-TDS dataset according to molecular function, biological process and Cellular component.

BLASTX analysis of 3062 unigenes showed 2185 total hits against NCBI non-redundant (nr) database with E value <1E-06. A majority (1.210; 55%) of top matches were from proteins of legume species, with maximum hits from *Glycine max *(528, 24% unigenes) and *Medicago truncatula *(338, 15% unigenes); only 6% (132 unigenes) matched with *Cicer arietinum*, indicating the novelty of the chickpea unigenes dataset. Among nonlegume species, majority of matches were with proteins of *Vitis vinifera *(275, 12% unigenes), *Ricinus communis *(214, 9% unigenes) and *Populus trichocarpa *(212, 9% unigenes). The availability of the whole genome and predicted proteins of these species and limited sequence information of legumes in the database may have led to the highest homology of chickpea sequences with these nonlegume genomes (Additional file 6). Functional annotation of unigenes by Blast2GO resulted in gene ontology functional classification terms for 2006 (92.0%) sequences, of which 1813 (90.3%) unigenes were functionally annotated (GO consensus and EC number) and 193 sequences were mapped but not annotated (Figure 4). At the second level GO, 1375 sequences were assigned to the biologic process category, 1422 sequences to the molecular function category, and 1311 sequences to the cellular component category (Figure 5). In biologic processes, "cellular process" and "metabolic process" was the most dominant term (27.2% of sequences), followed by "metabolic processes" (27.0%). In the molecular function category, "binding" (41.8%) was the most dominant term, followed by "catalytic activity" (36.6%); in the cellular compartments category, "cell part" (42.91%) was the most represented term, followed by "membrane-bounded organelle" (29.34%) and "organelle part" (10.04%). Additional file 7 gives details on GO analyses of UG-TDS sets.

### Pathway classification of transcripts

Of the 1808 annotated sequences, 656 were annotated with 812 Enzyme Commission (EC) codes and mapped to 108 different KEGG pathways. Of the 108 pathways contained within the metabolism category (metabolic pathways), 46 were represented by 43.44% of the 656 unigenes. KEGG metabolic pathways well represented by unigenes were biosynthesis of plant hormones (44 enzymes), biosynthesis of phenylpropanoids (29 enzymes) and terpenoids and steroids (24 enzymes), biosynthesis of alkaloids derived from histidine and purine (25 enzymes) and from the shikimate pathway (24 enzymes), starch and sucrose metabolism (24 enzymes), and arginine and proline metabolism (10 enzymes). Several hormone pathways, such as of abscisic acid, ethylene, salicylic acid, and jasmonic acid, are involved in one or more environmental stresses, including drought stress and other abiotic stresses processes [38-42]. A representative KEGG map for biosynthesis of plant hormones is given in Additional file 8.

### Gene ontology (GO) enrichment analysis

**Figure 6:**
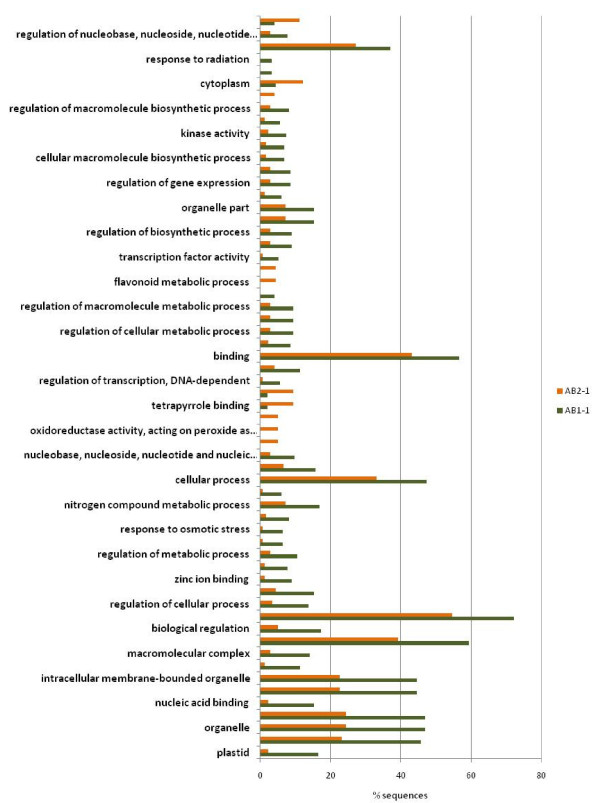
**Differential Gene Ontology terms between ESTs derived from ICC 4958 and ICC 1882 libraries under drought stress**. GO enrichment analysis between ESTs generated form AB1-1(forward subtracted library from the roots of ICC 4958 and ICC 1882) and AB2-1(reverse subtracted library from the roots of ICC 4958 and ICC 1882) SSH libraries using Fisher's exact test with a false discovery rate (FDR) cutoff of p ≤ 0.05. The numbers of transcripts associated with a specific GO term are represented as percentage of functionally annotated EST in their respective libraries.

Identification of overrepresented and underrepresented GO terms from a given list of genes from different libraries may help elucidate the functional relevance of these genes under drought stress. GO enrichment analysis found that 60 GO terms were differentially represented between AB1-1 and AB2-1 (Figure 6; additional file 9): 50 were overrepresented and 10 underrepresented in AB1-1. Several overrepresented terms were associated with stress response properties such as response to salt stress, osmotic stress, abiotic stimulus, radiation, and light stimulus. GO terms related to the flavonoid pathway (e.g., flavonoid metabolic process and flavonoid biosynthetic process) and peroxidase activity (e.g., oxidoreductase activity, acting on peroxide as acceptor and peroxidase activity) were underrepresented. The underrepresentation of these GO terms suggests downregulation of the flavonoid biosynthetic process and peroxidase activity under drought stress in roots of ICC 4958. Similar results have been reported in barley, chickpea, and mangrove under abiotic stress [7,43,44].

**Figure 7:**
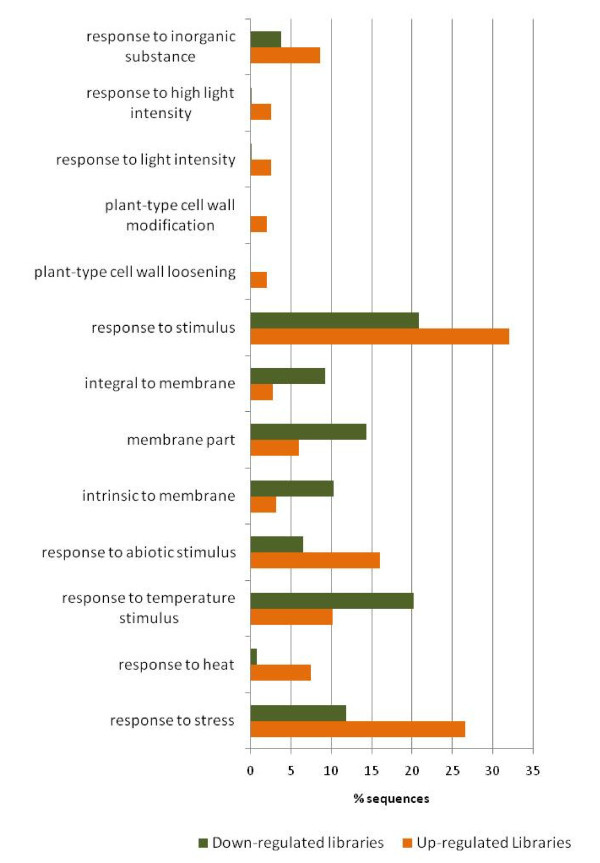
**Differential Gene Ontology terms between up regulatory and down regulatory transcript under drought stress**. GO enrichment analysis between ESTs generated from up regulated SSH library, AS1-1(forward subtracted library from the shoots of drought tolerant genotype, ICC 4958) and AR1-1(forward subtracted library from the roots of drought tolerant genotype, ICC 4958)) and down regulated SSH libraries, AS2-1(reverse subtracted library from the shoots of drought tolerant genotype, ICC 4958) and AR2-1(reverse subtracted library from the roots of drought tolerant genotype, ICC 4958) using Fisher's exact test with a false discovery rate (FDR) cutoff of p ≤ 0.05. The numbers of transcripts associated with a specific GO term are represented as percentage of functionally annotated EST in their respective libraries.

GO enrichment analysis was also performed between ESTs derived from the parental genotype library and RILs library to determine differential responses between parents and RILs. Compared with parental genotype libraries, 13 GO terms were significantly overrepresented in RILs bulk libraries (Additional file 10). GO enrichment analysis of forward-subtracted and reverse-subtracted SSH libraries to determine differential GO representation between up- and downregulated EST sets (Figure 7; Additional file 9) showed overrepresentation of GO terms related to stress response properties, such as response to stress, heat, temperature, and abiotic stimulus in the upregulated libraries (AS1-1 and AR1-1). Three GO terms intrinsic to membrane, membrane part, and integral to membrane were underrepresented in the upregulated libraries. These differential enriched GO terms related to stress response in upregulated libraries indicate the efficiency of the SSH technique to clone up- and downregulated genes by the forward- and reverse-subtraction methodology. By this analysis, we have a priori-defined gene networks involved in drought stress in chickpea, which can be used to select drought-responsive candidate genes in chickpea.

### Differential expression analysis of unigenes under drought stress

**Figure 8:**
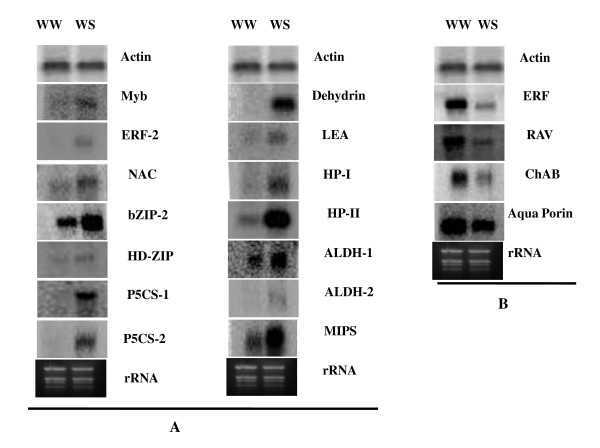
**Northern blot analysis of selected stress responsive genes**. Northern blot analysis showing expression of selected stress responsive ESTs (Myb, ERF-2, NAC, bZIP, HD-ZP, P5CS-1, P5CS-2, dehydrin, LEA, hypothetical protein 1, hypothetical protein-2, ALDH-1, ALDH-2, MIPS, ERF-1, RAV, chlorophyll a/b-binding protein, and aquaporin.) in well watered (WW) and water stressed (WS) ICC 4958 plants. ESTs are listed according to their annotation generated in present work. Chickpea actin cDNA and 28S ribosomal RNA were used as controls. Panel (A) and (B) show up-regulated and down regulated genes during drought stress, respectively.

Myoinositol-1-phosphate synthase (MIPS) and pyrroline-5-carboxylate synthetase (P5CS) (involved in the synthesis of pinitol and proline, respectively) were upregulated under drought stress (Figure 8). The concentration of pinitol, a cyclic sugar alcohol, is high in halophytic plants and plants adapted to drought [45]. MIPS transcript abundance, and it's content increases in several plant species in response to environmental stresses [27,46,47]. Two MIPS genes from chickpea *CaMIPS1 *and *CaMIPS2 *have been isolated and characterized for their role in water stress [48]. Differential patterns of MIPS-coding genes occur in maize [49], *Arabidopsis *[50], and rice [46]. Unigenes P5CS1 (UG-TDS_Contig353) and P5CS2 (HO066525) were significantly upregulated under water stress (Figure 8). A significant increase in proline concentrations has been reported in response to water stress in plants and accumulation of proline is considered as an indicator of stress-adaptive response of plants [51].

In our study, different LEA groups of genes were found in UG-TDS: 2 unigenes encoding HVA protein (HO065000, unigene_Contig11), 5 encoding LEA proteins (HO063258, HO065296, HO0065083, UG-TDS_Contig311 and UG-TDS_Contig524), 6 encoding dehydrin (UG-TDS_Contig232, Contig320, Contig622, UG-TDS HO064933, UG-TDS HO065247 and UG-TDS HO066163), and 1 encoding ERD proteins (HO065032). Among these LEA group 2 members [LEA (HO063258) and dehydrin (HO065247)] were found highly up regulated in drought stress (Figure 8). Earlier studies in chickpea have also reported the induction of LEA proteins under drought stress [27,34]. The expression profile of LEA genes under stress supports the role of LEA proteins as protective molecules that enable cells to survive protoplasmic water depletion [52]. Studies on overexpression of LEA genes also support the protective role of LEA proteins by improving the stress tolerance of transgenic plants. Expression of the barley gene *HVA1 *in wheat and rice increases drought tolerance [53], and overexpression of wheat LEA genes *PMA80 *and *PMA1959 *increases dehydration tolerance in transgenic rice [54].

Different members of aquaporins subfamilies were found in UG-TDS: which includes, 6 unigenes encoding plasma membrane intrinsic protein (UG-TDS HO062890, HO064502, HO064741, HO064425, HO064603 and HO064612), 5 unigenes encoding tonoplast intrinsic protein (UG-TDS HO064719, HO064351, UG-TDS_Contig278, UG-TDS_Contig19 and UG-TDS_Contig156) 2 unigenes encoding NOD26-like intrinsic protein (UG-TDS HO066903 and HO062732). The maximum numbers of the unigenes encoding aquaporin were found in root libraries and downregulation of one of the member (HO062890) under drought stress was conformed in northern blot analysis (Figure 8). This is similar to downregulation of transcripts and reduction in protein levels of most the *Arabidopsis *aquaporin genes under drought condition [55], which may be an adaptive strategy for plants to minimize water flow through cell membranes and uphold leaf turgor to minimize water loss. In tobacco, *NtPIP1.1 *and *NtPIP2.1 *expression is downregulated to reduce osmotic hydraulic conductance in the roots under drought stress [56], supporting the role of aquaporins in drought stress maintenance.

Eleven chickpea unigenes from UG-TDS were classified as members of the AP2/ERF superfamily: 10 under the ERF family and 1 under the RAV family. Three members of this family (ERF1, ERF-2, and RAV) were analyzed by Northern blot under drought stress conditions. ERF1 was downregulated whereas ERF2 was upregulated under stress conditions. Biosynthesis of ethylene and regulation of its activation pathway are important to mediate plant developmental processes and stress responses in plants [57,58]. The AP2/ERF family of transcription factors, especially the CBF/DREB and ERF subfamily, has been extensively studied in response to drought stress [59]. *CAP2*, a member of the chickpea AP2 family, is responsive to various abiotic stress and its overexpression in tobacco increases the tolerance to dehydration and salt stress [60]. Northern blot analysis showed that UG-TDS HO066286 coding for RAV (related to ABI3/VP1) transcription factor was downregulated under drought stress (Figure 8). *Arabidopsis *RAV1 is a brassinosteroid (BR) down-regulated gene. High level of BR is accompanied by a very low level of *RAV1 *transcripts and vice versa [61]. The involvement of BR pathway in the enhancement of tolerance to chilling, thermo, salt, mild drought injury and pathogen attack has been confirmed in several studies [62,63]. Therefore, the down regulation of RAV during terminal drought stress in our study may indicate the involvement of the BR pathway.

In chickpea, 3 members of the NAC gene family (*CarNAC1, CarNAC3 *and *CarNAC5*) are strongly induced by drought, salt, cold, and wounding [64]. We have identified 8 new members of this TF family in UG-TDS with one NAC gene (HO067315) that have increased expression under drought stress validated by northern blot result (Figure 8). Expression profiling and overexpression analysis of NAC genes in several plants supports their involvement in stress tolerance [65-67,11] K. Nakashima, L.P. Tran, D.V. Nguyen, M. Fujita, K. Maruyama, D. Todaka, Y. Ito, N. Hayashi, K. Shinozaki and K. Yamaguchi-Shinozaki, Functional analysis of a NAC-type transcription factor OsNAC6 involved in abiotic and biotic stress-responsive gene expression in rice, *Plant J*. **51 **(2007), pp. 617-630. **Full Text **via CrossRef | View Record in Scopus | Cited By in Scopus (50).

The HDZip gene (HO062575) was among the up regulated transcription factor, as reflected from northern blot results. Two members of this gene family (HO062575 and UG-TDS_Contig226) have been identified form UG-TDS. The functional information available on plant HDZIP genes suggest that at list some of these genes are involved in response to different environmental conditions [68]. Overexpression of sunflower HD-Zip gene *Habt-4 *confers drought tolerance in *Arabidopsis *[69], this is suggestive of important role of HD-Zip protein in regulation of expression of genes involved in drought tolerance.

Hypothetical proteins are genes of unknown functions predicted from the *Arabidopsis *or rice genome sequence. Two such genes HP-1 and HP-2 were significantly induced in WS plants. Several hypothetical genes have now been characterized by advanced bioinformatics tools by identifying similarity of conserved function domains. For example, in *Arabidopsis*, the family of BAG proteins initially annotated as hypothetical proteins are now annotated as *bag *gene family members, their function as regulators of apoptosis-like processes has also been characterized.

Functional characterization of such unknown hypothetical proteins can shed light on the mechanism of drought adaptation in chickpea.

We found transcript levels of the chlorophyll a/b-binding protein to be downregulated during stress. Most of the strongly downregulated transcripts were related to photosynthesis, photorespiration, and metabolism of amino acids and carbohydrates. In a dehydration shock treatment, the transcript level of chlorophyll a/b-binding protein remained unchanged [27]; similarly, in barley, chlorophyll a/b-binding protein transcript (NP_917525) levels do not change under dehydration shock treatment but are downregulated by drought stress treatment [70], indicating differential response of genes under dehydration and drought stress.

### Comparative transcript profiles of ICC 4958 and ICC 1882 under drought stress

**Figure 9:**
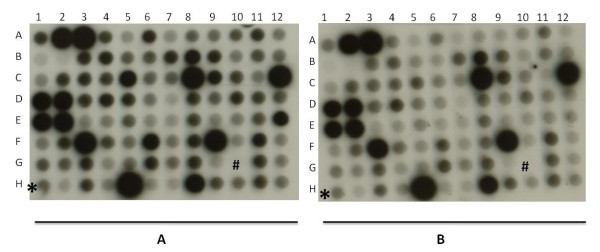
**A typical representative macroarray hybridization of SSH cDNA clones**. identical nylon membranes containing cDNA spots from subtracted cDNA library of chickpea were hybridized with α^32^P-dCTP labeled cDNA probes synthesized from WS ICC 4958 plants (A) and WS ICC 1882 plants (B). Actin (*) was used as internal control to normalize the signals of two different blots and NPTII (#) used as negative control to subtract the background noise.

To identify differentially regulated transcripts in response to terminal drought stress between drought-tolerant ICC 4958 and drought-susceptible ICC 1882, SSH libraries AB1-1 and AB2-1 were constructed. To validate these differentially expressed transcripts, a nylon macroarray, using unigenes from AB1-1 and AB2-1 libraries, was constructed. Total RNA from water-stressed ICC 4958 and ICC 1882 was used to assess the genotype-specific response of these genes under drought stress (Figure 9). The unigenes showing at least 1.5-fold of induction were selected for further analysis (additional file 11).

**Figure 10:**
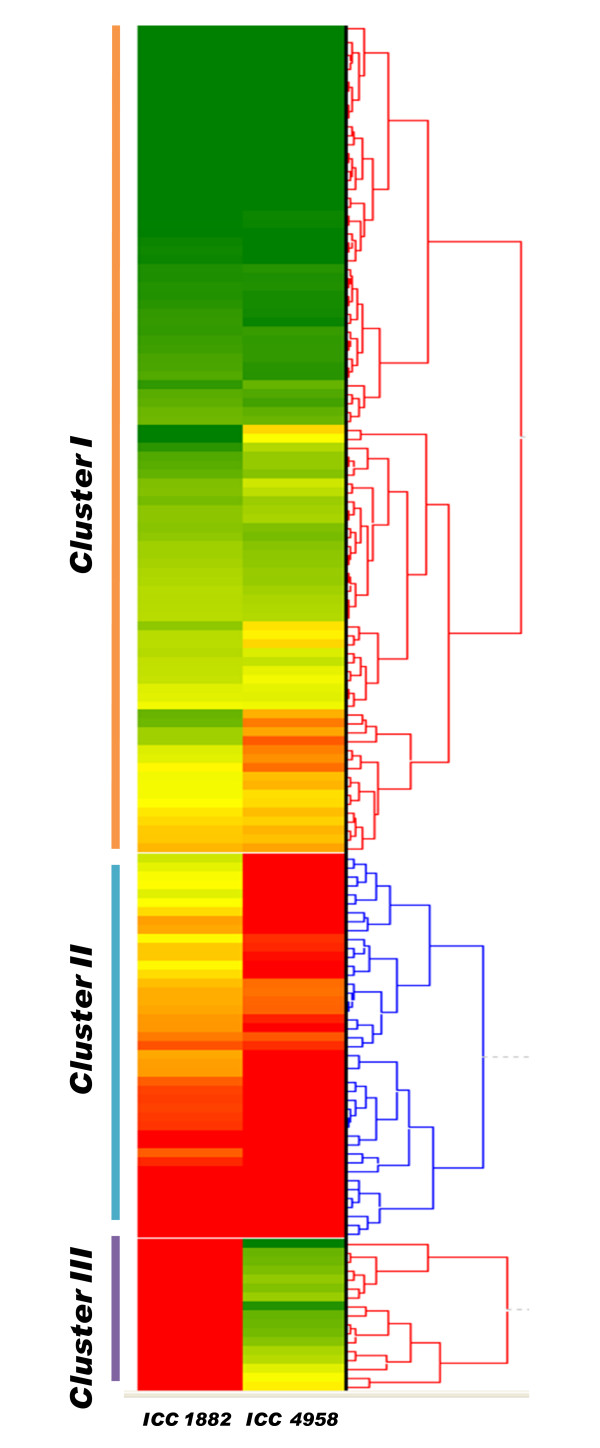
**Heat map of expression values of drought responsive genes in ICC 4958 and ICC 1882 under TDS**: Hierarchical clustering (average linkage and Euclidean distance matrix with the minimum similarity of 0.5) were performed using HCE version 2.0 beta web tool. Clustering of unigenes based on normalized signal intensity into three clusters (I, II, and III). The dendrogram of the array experiments reflects the similarity of the unigenes with respect to their gene-expression pattern. In the heat map red represents normalized expression values greater than the mean, green colour represents expression less than the mean and colour intensities in between the two represent the magnitude of the deviation from the mean. Colour scale (from green to red) represents the range of expression level.

**Figure 11:**
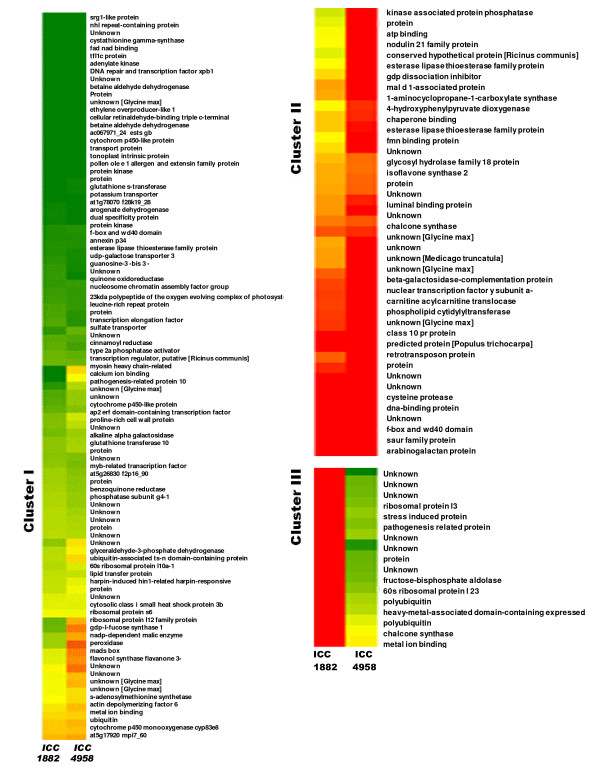
**Expanded portion of the heat map of Figure 10 depicting identities of the genes from each cluster**. Cluster I and II contains gene up regulated in ICC 4958 and cluster III contains genes up regulated in ICC 1882.

The normalized expression intensities of unigenes and the results of hierarchical clustering analysis according to their relative expression patterns is graphically represented by a heat map in Figure 10. Hierarchical clustering resulted in the formation of 3 clusters (cluster I, II and III). Clusters I and II included unigenes that were upregulated in ICC 4958, whereas cluster III included unigenes downregulated in ICC 4958 as compared with ICC 1882 (Figure 11).

Genes in clusters I and II were associated with metabolic process [e.g., ethylene biosynthesis (HO062211, HO062180), flavonoid synthesis (HO062384), and amino acid biosyntheses (HO062526, HO062310 and HO062183)] and also these genes shown to be involved in drought response in several other plants [71,72]. Upregulation of genes involved in ion binding and transport activities [e.g., ATP-binding proteins (HO063146), lipid transfer proteins (HO062394, HO062798), UDP-galactose transporters (HO062219), metal ion binding (HO062399), sulfate transporters (HO063202), tonoplast intrinsic proteins (HO062783), were also upregulated in ICC 4958. In an earlier study, we reported by *in silico *differential expression analysis the upregulation of the tonoplast intrinsic protein in the roots of ICC 4958, which mediates the regulation of root hydraulic conductivity in response to environmental stimuli [9]. Several stress-related genes [e.g., pathogenesis-related proteins (HO062911, HO062939) and peroxidase (HO062698), chaperone binding (HO062569) and small heat shock protein (HO062866)] upregulated in ICC 4958 and have been shown to be induced by wounding, salt, and cold stress in other plant species [73,74] indicates multiple stress induction of these genes. Similarly known stress-responsive transcription factors and regulators such as the AP2/ERF domain-containing transcription factor (HO062802), MYB transcription factor (HO062363), DNA repair and transcription factor XPB1 (HO062308), and transcription regulators (HO062392) were also upregulated in ICC 4958. A similar differential induction of these genes or gene categories in drought-tolerant genotypes in response to drought stress during the reproductive stage has been reported in barley [75].

Cluster III contained unigenes that were upregulated in ICC 1882 but not ICC 4958. One upregulated unigene encoded fructose-bisphosphate aldolase (HO063129), whose downregulation could inhibit gluconeogenesis for conserving energy in drought-stressed plants (41).

**Figure 12:**
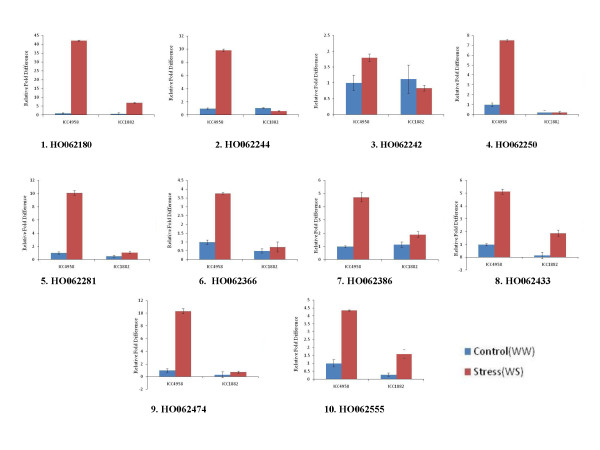
**Comparative expression analysis of selected unigenes between ICC 4958 and ICC 1882 chickpea genotypes in response to drought stress**. Relative expression levels (fold difference) of 10 selected unigens in ICC 4958 and ICC 1882 chickpea genotypes under terminal drought stress were evaluated using qPCR analysis. Error bars represent Standard error of the mean (Number of replication n = 3). Unigenes used for qPCR analysis were: 1-aminocyclopropane-1-carboxylate synthase (**HO062180**), esterase lipase thioesterase family protein-1(**HO062244**), yippee family protein (Putative zinc binding protein) (**HO062242**), calcium ion binding (**HO062250**), protein kinase (**HO062281**), MADS box protein (**HO062366**), esterase lipase thioesterase family protein-2 (**HO062386**), alkaline alpha galactosidase (**HO062433**), leucine-rich repeat protein (**HO062474**) and GDP dissociation inhibitor (**HO062555**).

To validate the results of dot blot analysis, 10 differentially expressed unigenes were analyzed by qPCR. Real-time PCR confirmed the differential expression of these genes under terminal drought stress conditions (Figure 12). The genes showing significant differential expression between the 2 genotypes can be explored as potential candidate genes that can confer terminal drought tolerance in chickpea, using transgenic overexpression and TILLING (targeting induced local lesions in genomes) analysis.

## Conclusions

We report the sequencing, assembly, and annotation of 5494 high-quality drought-responsive EST sequences from chickpea. This dataset was generated from SSH libraries constructed using drought-tolerant and -susceptible chickpea genotypes and bulks of their progenies exhibiting HRB and LRB phenotypes. SSH libraries allowed cloning genes that are specifically up- and downregulated from the roots and shoots of chickpea in response to terminal drought. Moreover, we identified more than 1500 novel unigenes in chickpea that are associated with terminal drought stress. Besides several transcripts coding for known stress-related proteins, several novel genes with unknown functions that may have a potential role in drought tolerance in chickpea were also identified. This study also provides a comparative overview of genotype-specific expression patterns of more than 830 unigenes in root tissues of chickpea in response to drought. The up- and downregulation of some unigenes was confirmed by real-time qPCR. The EST dataset and the information about transcription of several genes can be useful for the research community and help identify potential candidate genes for drought tolerance in chickpea. Our study can also serve as an important resource for developing functional markers, full-length gene isolation, TILLING, drought-responsive promoter isolation, and in drought functional genomic studies involving overexpression, e-QTL, and manipulation of drought tolerance in chickpea.

## Authors' contributions

RS, RV, PKJ, SMK and AAD planned the experiments. VV, AAD and VK were involved in setting up drought experiments and isolation of RNA. AAD, VK were involved in cloning and sequencing of cDNA SSH libraries, dot blot, northern blot and real time PCR experiments. AAD, NLR and RV were involved in bioinformatics analysis. AAD, RS and RV analyzed the experiments. AAD and RS prepared the manuscript. All authors read and approved the final manuscript.

## Supplementary Material

Additional file 1 **Summary of earlier work done towards identifying ESTs associated with drought stress in chickpea**. Click here for file

Additional file 2 **Primer sequences for qPCR analysis**. All primer sequences used for qPCR analysis in the manuscript are listed. Click here for file

Additional file 3 **Daily NTR ratio of each well watered (WW) and water stressed (WS) ICC 4958, ICC 1882 and RILs**. **(A) **Change in NTR ratio of well watered (WW) and water stressed (WS) ICC 4958 and ICC 1882 plants. **(B) **Change in NTR ratio of high root biomass and low root biomass RILs along with parental lines under water stressed (WS) condition. Click here for file

Additional file 4 **Graphical representation of Chickpea unigene assembly UG-TDS**. (a) Distribution of chickpea EST members in contigs after the assembly process. (b) Distribution of contigs according to the EST numbers. Each contig categories represents number of ESTs per contig. Green bars indicate the EST size and the blue bars indicate number of contigs belonging to respective EST size categories. Click here for file

Additional file 5 **UG-TDS BLASTN analysis results**. Table showing BLASTN analysis results of UG-TDS dataset with EST datasets of EST datasets of Mt (*Medicago Truncatula*), Gm (*Glycine max*), Pv (*Phaseolus vulgaris*), Lj (*Lotus japonicus*), Pa (*Populus alba*), Os (*Oryza Sativa*) and At (*Arabidopsis thaliana*) with corresponding details of GB ID numbers, descriptions and E-value. Click here for file

Additional file 6 **BLASTX similarity search of the UG-TDS against the NCBI non-redundant protein database**. (A) Distribution of top matches against the NCBI taxonomic domains. (B) Distribution of e-value scores. Click here for file

Additional file 7 **Functional annotation of UG-TDS results**. Table showing functional categorization results of UG-TDS dataset using Blast2go tool. Table represent corresponding details of sequence description of BLASTX hit, E-values, *Gene Ontology terms *and Enzyme Commission entries. Click here for file

Additional file 8 **KEGG pathway for Biosynthesis of plant hormones**: 78 differentially expressed unigenes under drought stress were identified as a candidates involves in different plant hormones such as Jasmonic acid, ethylene and salicylic acid and gibberellin. Click here for file

Additional file 9 **GO enrichment analysis using GOSSIP module of BLAST2GO program**. *Table S1*: Results of GO enrichment analysis done using transcripts generated from AB1-1 library as test set and AB2-1 as reference set with the FDR filter value 0.05. The 60 GO terms were differentially represented in these two libraries. Out of then 50 were over represented and 10 were under represented. *Table S2*: Results of GO enrichment analysis done using transcripts generated from bulks of RILs as test set and SSH unigenes from individual parental libraries as reference set with the FDR filter value 0.05. The 13 GO terms were over represented in libraries from bulk of RILs. *Table S3*: Results of GO enrichment analysis done using transcripts generated from up regulated libraries (AS1-1 and AR1-1) as test set and unigenes from down regulatory libraries (AS2-1 and AR2-1) as reference set with the FDR filter value 0.05. The 10 Go terms were overrepresented in up regulated libraries and three GO terms were under represented. Click here for file

Additional file 10 **Differential Gene Ontology terms between parental line (ICC 4958 & ICC 1882) and bulks of RILs under drought stress**. GO enrichment analysis between ESTs generated from parental line (From AS and AR libraries) and ESTs form bulks of RILs using Fisher's exact test with a false discovery rate (FDR) cutoff of p ≤ 0.05. The numbers of transcripts associated with a specific GO term are represented as percentage of functionally annotated EST in their respective libraries Click here for file

Additional file 11 **Genotype specific response of chickpea unigenes in response to terminal drought stress**. Expression profiling of differentially expressed ESTs generated by SSH libraries were analysed in drought stressed ICC 4958 and ICC 1882 using dot-blot expression analysis. Differential responses of unigenes are represented in normalised signal intensities values. Standard deviations are calculated from three different experiments. Signal intensity of Actin (GenBank: EU529707) used for normalisation of the signals between the blots and NPTII was used for signal background correction. Unigenes are listed according to their annotation generated in present work. Click here for file
