# Supplementary material for: Comparative analysis of expressed sequence tags (ESTs) between drought-tolerant and -susceptible genotypes of chickpea under terminal drought stress
Source: BMC Plant Biol. 2011 Apr 22;11:70. doi: 10.1186/1471-2229-11-70 (PMC3110109; doi:10.1186/1471-2229-11-70)
Supplement: Additional file 1 — Summary of earlier work done towards identifying ESTs associated with drought stress in chickpea. [file 1471-2229-11-70-S1.DOCX]

|  | **Genotype** | **Type of tissue** | **Conditions** | **Stage** | **Type of cDNA library** | **No of ESTs generated** | **No of unigenes** | **Validation of expression** | **References** |
| --- | --- | --- | --- | --- | --- | --- | --- | --- | --- |
| 1 | ICC 4958, ICC 1882 and RIL lines | Shoot and root | Drought stress (Dry down experiment at flowering stage) | Flowering | SSH | 6053 | 3062 | Dot blot, Northern analysis and qPCR | Present study |
| 2 | BGD72 and ICCV2 | Seedling | Dehydration (withholding water for 3, 6, and 12 days) | Seedling | SSH | 2700 | 319 | cDNA macroarray and Northern analysis | Jain et al, 2010 |
| 3 | ICC 4958, ICC 1882, JG 11 and ICCV 2 | Root | Drought and salinity (Dry down and field drought stress) | Flowering | cDNA(non normalised) | 20,162 | 6,404 |  | Varshney et al, 2009 |
| 4 | ILC 588 | Root | Dehydration (6 hr) | Seedling | Super SAGE | 80,238  (26 bp tags) | 17,493 | qPCR | Molina et al., 2008 |
| 5 | cv. Xj-209 | Seedling | PEG 4000 | Seedling | cDNA (non normalised) | 5097 | 2955 | qPCR | Gao et al., 2008 |
| 6 | BG 1103, BG 362, Kaniva and Genesis 508 | Shoot and  root | Drought stress |  |  |  |  | Microarray and qPCR.  (pulse microarray was developed from chickpea and grasspea ESTs available in public domain) | Mantri et al., 2007 |
| 7 | ICC 4958 and Annegiri | Root | Control condition | Flowering | SSH | 2858 | 477 |  | [Buhariwalla et al., 2005](http://www.ncbi.nlm.nih.gov/pubmed?term=%22Buhariwalla%20HK%22%5BAuthor%5D) |
| 8 | BGD72 | Seedling | Dehydration (withholding water for 5 hr) | Seedling | SSH | 377 (only differentially induced clones were sequenced) | 101 | Dot blot and Northern analysis | Boominathan et al., 2004 |
| 9 | Castellana | Seedling | PEG 4000 | Seedling | cDNA  (non normalised) | 47 differentially induced clones after screenings |  | Northern analysis | Romo et al., 2001 |

**Additional File1:**
