# Supplementary material for: Comparative analysis of expressed sequence tags (ESTs) between drought-tolerant and -susceptible genotypes of chickpea under terminal drought stress
Source: BMC Plant Biol. 2011 Apr 22;11:70. doi: 10.1186/1471-2229-11-70 (PMC3110109; doi:10.1186/1471-2229-11-70)
Supplement: Additional file 3 — Daily NTR ratio of each well watered (WW) and water stressed (WS) ICC 4958, ICC 1882 and RILs. (A) Change in NTR ratio of well watered (WW) and water stressed (WS) ICC 4958 and ICC 1882 plants. (B) Change in NTR ratio of high root biomass and low root biomass RILs along with parental lines under water stressed (WS) condition. [file 1471-2229-11-70-S3.DOCX]

**Additional files 3.**

**Figure 1: (A)** Change in NTR ratio of well water (WW) and water stressed (WS) ICC 4958 and ICC 1882 plants. **(B)** Change in NTR ratio of high root biomass and low root biomass RILs along with parental lines under water stressed (WS) condition**.**

**A.**

**B.**

|  | **ICC4958** |  | **ICC4958** |  | **ICC1882** |  | **ICC1882** |  |
| --- | --- | --- | --- | --- | --- | --- | --- | --- |
|  | **WW** | **S.D.** | **WS** | **S.D.** | **WW** | **S.D.** | **WS** | **S.D.** |
| 1 Day | 1.002133 | 0.082338 | 0.968415 | 0.179177 | 0.993342 | 0.057882 | 0.936022 | 0.121943 |
| 2-Day | 1.003067 | 0.080345 | 1.030018 | 0.063184 | 0.995747 | 0.097794 | 0.978665 | 0.107671 |
| 3-Day | 0.9948 | 0.161139 | 1.001567 | 0.238167 | 1.010911 | 0.130275 | 1.085312 | 0.225961 |
| 4-Day | 0.999401 | 0.041073 | 0.971274 | 0.227365 | 0.979606 | 0.178442 | 1.098822 | 0.041001 |
| 5-Day | 1.005261 | 0.159075 | 0.864834 | 0.211843 | 0.975955 | 0.210698 | 1.046015 | 0.124756 |
| 6-Day | 1.003206 | 0.064262 | 0.955174 | 0.01402 | 0.976794 | 0.212622 | 0.940422 | 0.115141 |
| 7-Day | 1.000535 | 0.064193 | 0.999935 | 0.10283 | 0.980414 | 0.181046 | 0.89376 | 0.197365 |
| 8-Day | 0.996212 | 0.083613 | 0.867679 | 0.094543 | 0.976497 | 0.211271 | 0.620533 | 0.150207 |
| 9-Day | 0.998077 | 0.034985 | 0.611846 | 0.219027 | 0.978162 | 0.197677 | 0.249564 | 0.215204 |
| 10-Day | 1.0024 | 0.068199 | 0.459336 | 0.297114 | 0.990779 | 0.124306 | 0.20643 | 0.080093 |
| 11-Day | 1.001309 | 0.045486 | 0.369075 | 0.182097 | 0.977201 | 0.19909 | 0.23052 | 0.049774 |
| 12-Day | 1.005135 | 0.127476 | 0.161894 | 0.13118 | 0.969701 | 0.263824 | 0.087523 | 0.021056 |

**The NTR values of ICC 4958 and ICC 1882 well watered (WW) and water stressed (WS) experiments on respective day. The standard deviation (S.D.) was calculated from the mean of three replications.**
