# Supplementary figures and images for: Comparative analysis of expressed sequence tags (ESTs) between drought-tolerant and -susceptible genotypes of chickpea under terminal drought stress
Source: BMC Plant Biol. 2011 Apr 22;11:70. doi: 10.1186/1471-2229-11-70 (PMC3110109; doi:10.1186/1471-2229-11-70)

## Slide 1
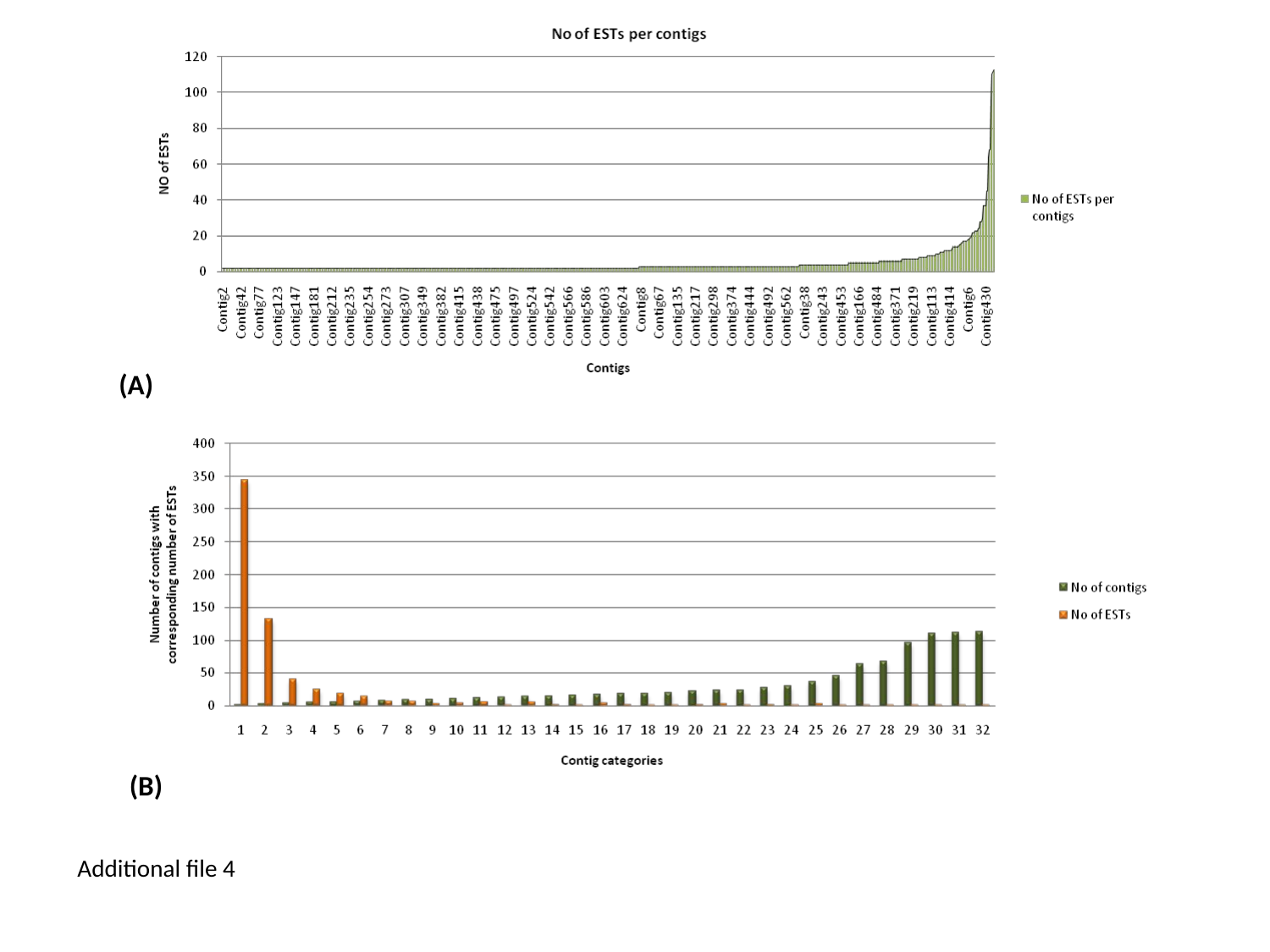

(A)
(B)
Additional file 4

Supplement: Additional file 4 — Graphical representation of Chickpea unigene assembly UG-TDS. (a) Distribution of chickpea EST members in contigs after the assembly process. (b) Distribution of contigs according to the EST numbers. Each contig categories represents number of ESTs per contig. Green bars indicate the EST size and the blue bars indicate number of contigs belonging to respective EST size categories. [file 1471-2229-11-70-S4.PPT]

## Slide 1
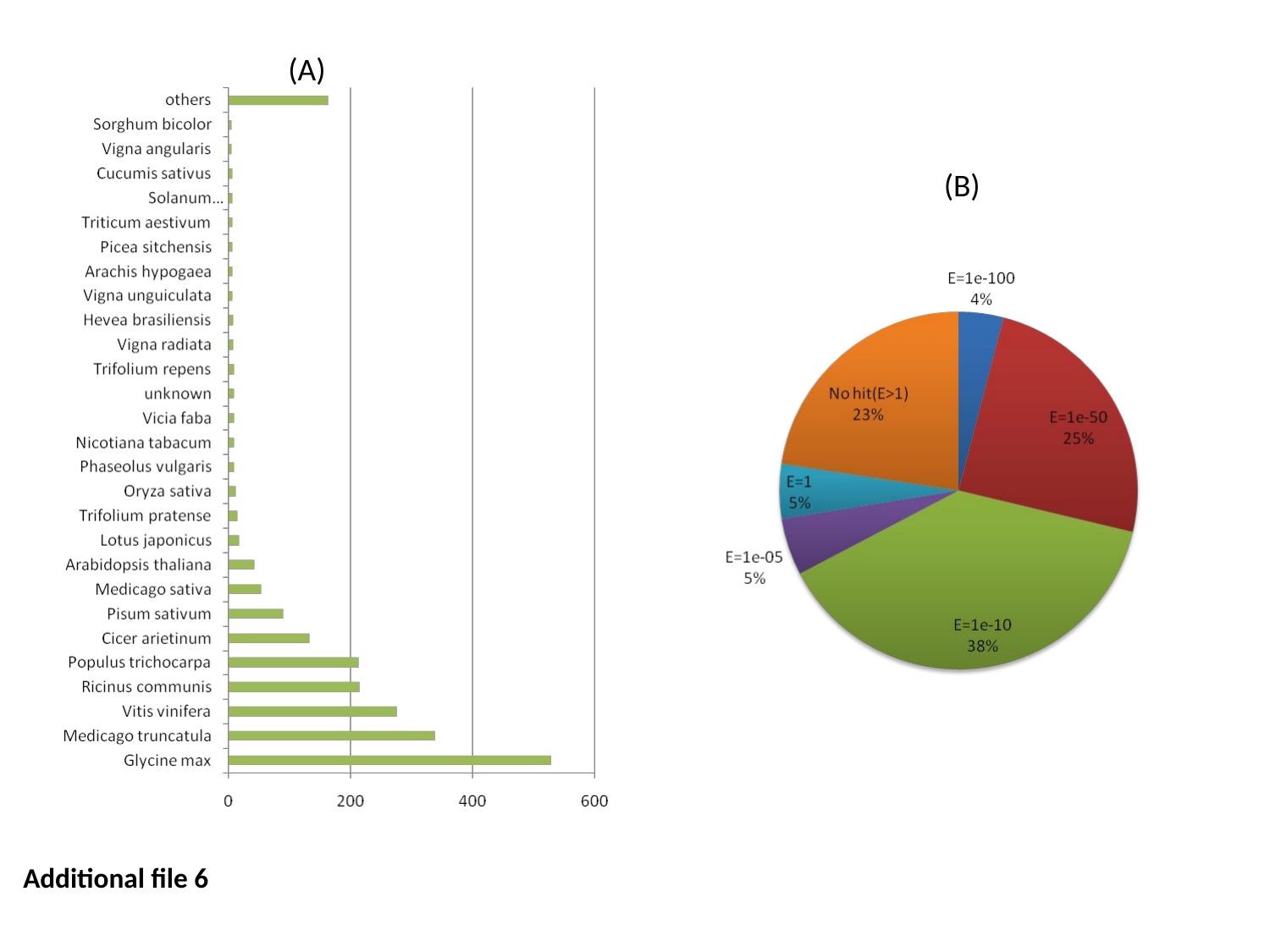

(A)
(B)
Additional file 6

Supplement: Additional file 6 — BLASTX similarity search of the UG-TDS against the NCBI non-redundant protein database. (A) Distribution of top matches against the NCBI taxonomic domains. (B) Distribution of e-value scores. [file 1471-2229-11-70-S6.PPT]

## Slide 1
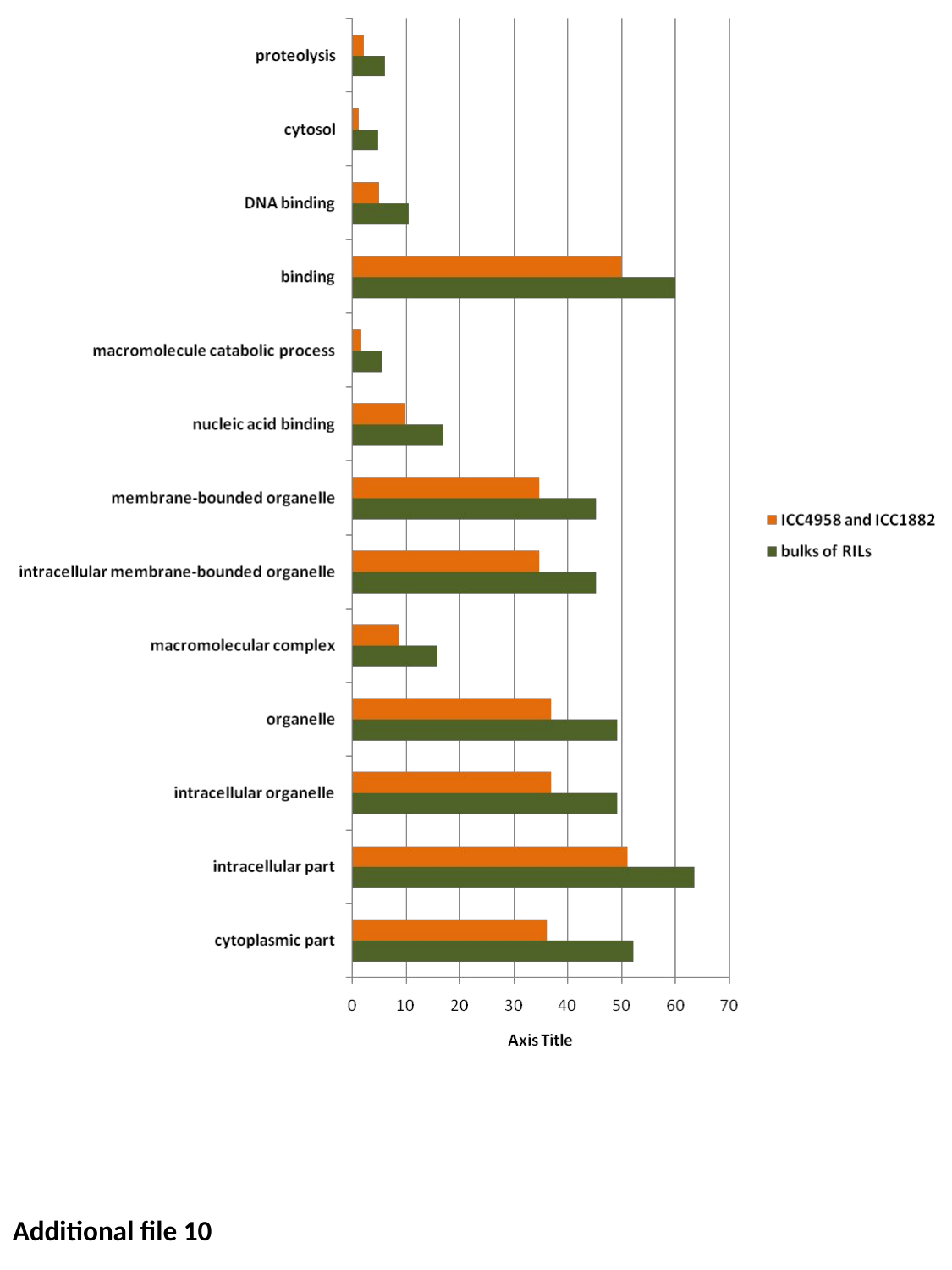

Additional file 10

Supplement: Additional file 10 — Differential Gene Ontology terms between parental line (ICC 4958 & ICC 1882) and bulks of RILs under drought stress. GO enrichment analysis between ESTs generated from parental line (From AS and AR libraries) and ESTs form bulks of RILs using Fisher's exact test with a false discovery rate (FDR) cutoff of p ≤ 0.05. The numbers of transcripts associated with a specific GO term are represented as percentage of functionally annotated EST in their respective libraries [file 1471-2229-11-70-S10.PPT]
