# Supplementary material for: Comparative analysis of expressed sequence tags (ESTs) between drought-tolerant and -susceptible genotypes of chickpea under terminal drought stress
Source: BMC Plant Biol. 2011 Apr 22;11:70. doi: 10.1186/1471-2229-11-70 (PMC3110109; doi:10.1186/1471-2229-11-70)
Supplement: Additional file 8 — KEGG pathway for Biosynthesis of plant hormones: 78 differentially expressed unigenes under drought stress were identified as a candidates involves in different plant hormones such as Jasmonic acid, ethylene and salicylic acid and gibberellin. [file 1471-2229-11-70-S8.PPT]

## Slide 1
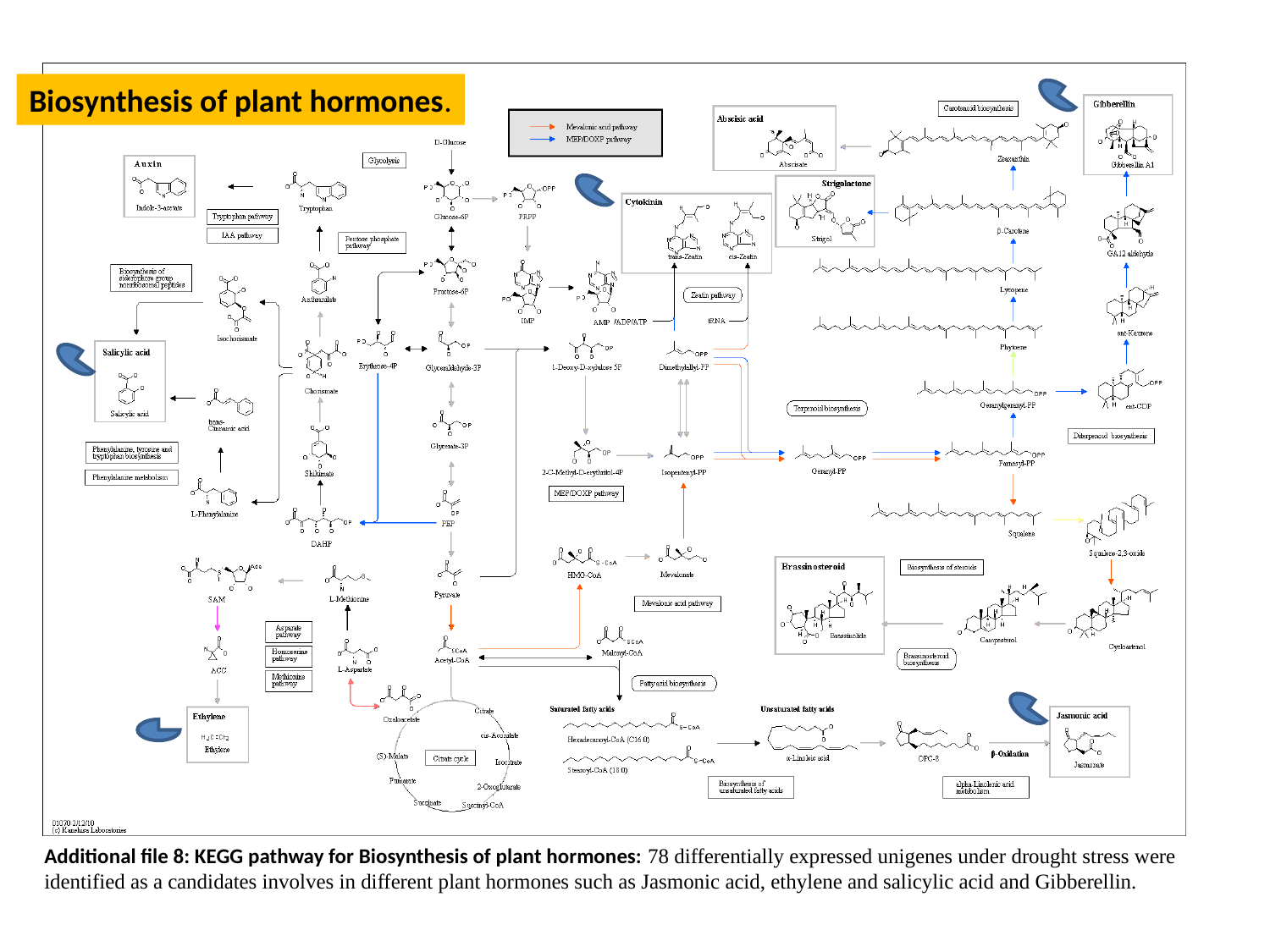

Biosynthesis of plant hormones.
Additional file 8: KEGG pathway for Biosynthesis of plant hormones: 78 differentially expressed unigenes under drought stress were identified as a candidates involves in different plant hormones such as Jasmonic acid, ethylene and salicylic acid and Gibberellin.
