# Supplementary material for: Comparative analysis of expressed sequence tags (ESTs) between drought-tolerant and -susceptible genotypes of chickpea under terminal drought stress
Source: BMC Plant Biol. 2011 Apr 22;11:70. doi: 10.1186/1471-2229-11-70 (PMC3110109; doi:10.1186/1471-2229-11-70)
Supplement: Additional file 9 — GO enrichment analysis using GOSSIP module of BLAST2GO program. Table S1: Results of GO enrichment analysis done using transcripts generated from AB1-1 library as test set and AB2-1 as reference set with the FDR filter value 0.05. The 60 GO terms were differentially represented in these two libraries. Out of then 50 were over represented and 10 were under represented. Table S2: Results of GO enrichment analysis done using transcripts generated from bulks of RILs as test set and SSH unigenes from individual parental libraries as reference set with the FDR filter value 0.05. The 13 GO terms were over represented in libraries from bulk of RILs. Table S3: Results of GO enrichment analysis done using transcripts generated from up regulated libraries (AS1-1 and AR1-1) as test set and unigenes from down regulatory libraries (AS2-1 and AR2-1) as reference set with the FDR filter value 0.05. The 10 Go terms were overrepresented in up regulated libraries and three GO terms were under represented. [file 1471-2229-11-70-S9.DOCX]

| **GO Term** | **Name** | **FDR** | **FWER** | **single test p-Value** | **# in test group**  **(AB1-1)** | **# in reference group (AB2-1)** | **# non annot test** | **# non annot reference group** | **Over/Under** |
| --- | --- | --- | --- | --- | --- | --- | --- | --- | --- |
| [GO:0009536](GossipInfo:GO:0009536) | plastid | 7.53E-06 | 2.69E-06 | 3.04E-07 | 42 | 4 | 214 | 177 | over |
| [GO:0044444](GossipInfo:GO:0044444) | cytoplasmic part | 7.53E-06 | 8.58E-06 | 8.78E-07 | 117 | 42 | 139 | 139 | over |
| [GO:0043226](GossipInfo:GO:0043226) | organelle | 7.53E-06 | 9.60E-06 | 9.75E-07 | 120 | 44 | 136 | 137 | over |
| [GO:0043229](GossipInfo:GO:0043229) | intracellular organelle | 7.53E-06 | 9.60E-06 | 9.75E-07 | 120 | 44 | 136 | 137 | over |
| [GO:0003676](GossipInfo:GO:0003676) | nucleic acid binding | 7.53E-06 | 1.21E-05 | 1.40E-06 | 39 | 4 | 217 | 177 | over |
| [GO:0043227](GossipInfo:GO:0043227) | membrane-bounded organelle | 7.53E-06 | 1.32E-05 | 1.49E-06 | 114 | 41 | 142 | 140 | over |
| [GO:0043231](GossipInfo:GO:0043231) | intracellular membrane-bounded organelle | 7.53E-06 | 1.32E-05 | 1.49E-06 | 114 | 41 | 142 | 140 | over |
| [GO:0009507](GossipInfo:GO:0009507) | chloroplast | 4.57E-05 | 9.14E-05 | 9.08E-06 | 29 | 2 | 227 | 179 | over |
| [GO:0032991](GossipInfo:GO:0032991) | macromolecular complex | 1.14E-04 | 2.82E-04 | 2.35E-05 | 36 | 5 | 220 | 176 | over |
| [GO:0044424](GossipInfo:GO:0044424) | intracellular part | 1.14E-04 | 2.85E-04 | 2.40E-05 | 152 | 71 | 104 | 110 | over |
| [GO:0065007](GossipInfo:GO:0065007) | biological regulation | 2.70E-04 | 7.43E-04 | 5.43E-05 | 44 | 9 | 212 | 172 | over |
| [GO:0044464](GossipInfo:GO:0044464) | cell part | 6.18E-04 | 0.0018517 | 1.16E-04 | 185 | 99 | 71 | 82 | over |
| [GO:0050794](GossipInfo:GO:0050794) | regulation of cellular process | 6.20E-04 | 0.0020141 | 1.18E-04 | 35 | 6 | 221 | 175 | over |
| [GO:0050789](GossipInfo:GO:0050789) | regulation of biological process | 7.47E-04 | 0.0026115 | 1.65E-04 | 39 | 8 | 217 | 173 | over |
| [GO:0008270](GossipInfo:GO:0008270) | zinc ion binding | 8.53E-04 | 0.0031921 | 1.89E-04 | 23 | 2 | 233 | 179 | over |
| [GO:0016209](GossipInfo:GO:0016209) | antioxidant activity | 0.0106721 | 0.0079724 | 3.18E-04 | 0 | 9 | 256 | 172 | under |
| [GO:0016684](GossipInfo:GO:0016684) | oxidoreductase activity, acting on peroxide as acceptor | 0.0106721 | 0.0079724 | 3.18E-04 | 0 | 9 | 256 | 172 | under |
| [GO:0004601](GossipInfo:GO:0004601) | peroxidase activity | 0.0106721 | 0.0079724 | 3.18E-04 | 0 | 9 | 256 | 172 | under |
| [GO:0046906](GossipInfo:GO:0046906) | tetrapyrrole binding | 0.0111621 | 0.0138563 | 5.17E-04 | 5 | 17 | 251 | 164 | under |
| [GO:0020037](GossipInfo:GO:0020037) | heme binding | 0.0111621 | 0.0138563 | 5.17E-04 | 5 | 17 | 251 | 164 | under |
| [GO:0009812](GossipInfo:GO:0009812) | flavonoid metabolic process | 0.0150529 | 0.0260003 | 7.90E-04 | 0 | 8 | 256 | 173 | under |
| [GO:0009813](GossipInfo:GO:0009813) | flavonoid biosynthetic process | 0.0150529 | 0.0260003 | 7.90E-04 | 0 | 8 | 256 | 173 | under |
| [GO:0003677](GossipInfo:GO:0003677) | DNA binding | 0.00389058 | 0.0154428 | 8.08E-04 | 20 | 2 | 236 | 179 | over |
| [GO:0019222](GossipInfo:GO:0019222) | regulation of metabolic process | 0.00553321 | 0.0232439 | 0.0012294 | 27 | 5 | 229 | 176 | over |
| [GO:0009651](GossipInfo:GO:0009651) | response to salt stress | 0.00555626 | 0.0260496 | 0.0012402 | 16 | 1 | 240 | 180 | over |
| [GO:0006970](GossipInfo:GO:0006970) | response to osmotic stress | 0.00555626 | 0.0260496 | 0.0012402 | 16 | 1 | 240 | 180 | over |
| [GO:0043234](GossipInfo:GO:0043234) | protein complex | 0.00778643 | 0.0381895 | 0.001845 | 21 | 3 | 235 | 178 | over |
| [GO:0006807](GossipInfo:GO:0006807) | nitrogen compound metabolic process | 0.00805482 | 0.0414125 | 0.0019325 | 43 | 13 | 213 | 168 | over |
| [GO:0016210](GossipInfo:GO:0016210) | naringenin-chalcone synthase activity | 0.034127 | 0.0659873 | 0.0019514 | 0 | 7 | 256 | 174 | under |
| [GO:0051252](GossipInfo:GO:0051252) | regulation of RNA metabolic process | 0.00817656 | 0.043982 | 0.0020489 | 15 | 1 | 241 | 180 | over |
| [GO:0009987](GossipInfo:GO:0009987) | cellular process | 0.00828498 | 0.0465296 | 0.0020891 | 121 | 60 | 135 | 121 | over |
| [GO:0005737](GossipInfo:GO:0005737) | cytoplasm | 0.0363399 | 0.0785252 | 0.0021237 | 11 | 22 | 245 | 159 | under |
| [GO:0034641](GossipInfo:GO:0034641) | cellular nitrogen compound metabolic process | 0.0103616 | 0.0612304 | 0.0027109 | 40 | 12 | 216 | 169 | over |
| [GO:0006139](GossipInfo:GO:0006139) | nucleobase, nucleoside, nucleotide and nucleic acid metabolic process | 0.0103616 | 0.0627213 | 0.0027668 | 25 | 5 | 231 | 176 | over |
| [GO:0006355](GossipInfo:GO:0006355) | regulation of transcription, DNA-dependent | 0.0122352 | 0.0797104 | 0.0033678 | 14 | 1 | 242 | 180 | over |
| [GO:0009628](GossipInfo:GO:0009628) | response to abiotic stimulus | 0.0122352 | 0.080824 | 0.0033772 | 29 | 7 | 227 | 174 | over |
| [GO:0005506](GossipInfo:GO:0005506) | iron ion binding | 0.0482627 | 0.113691 | 0.0034814 | 10 | 20 | 246 | 161 | under |
| [GO:0005488](GossipInfo:GO:0005488) | binding | 0.0122352 | 0.082497 | 0.0035067 | 145 | 78 | 111 | 103 | over |
| [GO:0016772](GossipInfo:GO:0016772) | transferase activity, transferring phosphorus-containing groups | 0.0122352 | 0.0849089 | 0.0035441 | 22 | 4 | 234 | 177 | over |
| [GO:0031323](GossipInfo:GO:0031323) | regulation of cellular metabolic process | 0.0123237 | 0.0939154 | 0.0041059 | 24 | 5 | 232 | 176 | over |
| [GO:0080090](GossipInfo:GO:0080090) | regulation of primary metabolic process | 0.0123237 | 0.0939154 | 0.0041059 | 24 | 5 | 232 | 176 | over |
| [GO:0060255](GossipInfo:GO:0060255) | regulation of macromolecule metabolic process | 0.0123237 | 0.0939154 | 0.0041059 | 24 | 5 | 232 | 176 | over |
| [GO:0003723](GossipInfo:GO:0003723) | RNA binding | 0.0148699 | 0.115492 | 0.0044191 | 10 | 0 | 246 | 181 | over |
| [GO:0003700](GossipInfo:GO:0003700) | transcription factor activity | 0.0182335 | 0.147673 | 0.0055056 | 13 | 1 | 243 | 180 | over |
| [GO:0031326](GossipInfo:GO:0031326) | regulation of cellular biosynthetic process | 0.0182335 | 0.151412 | 0.0060464 | 23 | 5 | 233 | 176 | over |
| [GO:0009889](GossipInfo:GO:0009889) | regulation of biosynthetic process | 0.0182335 | 0.151412 | 0.0060464 | 23 | 5 | 233 | 176 | over |
| [GO:0044446](GossipInfo:GO:0044446) | intracellular organelle part | 0.0190751 | 0.165826 | 0.0069263 | 39 | 13 | 217 | 168 | over |
| [GO:0044422](GossipInfo:GO:0044422) | organelle part | 0.0190751 | 0.165826 | 0.0069263 | 39 | 13 | 217 | 168 | over |
| [GO:0090304](GossipInfo:GO:0090304) | nucleic acid metabolic process | 0.0254327 | 0.223833 | 0.0081141 | 15 | 2 | 241 | 179 | over |
| [GO:0010468](GossipInfo:GO:0010468) | regulation of gene expression | 0.0254327 | 0.229623 | 0.0088326 | 22 | 5 | 234 | 176 | over |
| [GO:0051171](GossipInfo:GO:0051171) | regulation of nitrogen compound metabolic process | 0.0254327 | 0.229623 | 0.0088326 | 22 | 5 | 234 | 176 | over |
| [GO:0034645](GossipInfo:GO:0034645) | cellular macromolecule biosynthetic process | 0.0288174 | 0.26659 | 0.0101822 | 17 | 3 | 239 | 178 | over |
| [GO:0009059](GossipInfo:GO:0009059) | macromolecule biosynthetic process | 0.0288174 | 0.26659 | 0.0101822 | 17 | 3 | 239 | 178 | over |
| [GO:0016301](GossipInfo:GO:0016301) | kinase activity | 0.0300737 | 0.281879 | 0.0117189 | 19 | 4 | 237 | 177 | over |
| [GO:0030529](GossipInfo:GO:0030529) | ribonucleoprotein complex | 0.0309685 | 0.294989 | 0.0126004 | 14 | 2 | 242 | 179 | over |
| [GO:0010556](GossipInfo:GO:0010556) | regulation of macromolecule biosynthetic process | 0.0309685 | 0.299873 | 0.0127933 | 21 | 5 | 235 | 176 | over |
| [GO:0009416](GossipInfo:GO:0009416) | response to light stimulus | 0.0370053 | 0.3589 | 0.013246 | 8 | 0 | 248 | 181 | over |
| [GO:0009314](GossipInfo:GO:0009314) | response to radiation | 0.0370053 | 0.3589 | 0.013246 | 8 | 0 | 248 | 181 | over |
| [GO:0044237](GossipInfo:GO:0044237) | cellular metabolic process | 0.047316 | 0.440356 | 0.0176502 | 95 | 49 | 161 | 132 | over |
| [GO:0019219](GossipInfo:GO:0019219) | regulation of nucleobase, nucleoside, nucleotide and nucleic acid metabolic process | 0.0479931 | 0.451634 | 0.0183646 | 20 | 5 | 236 | 176 | over |

Additional file 9; Table S1: Results of GO enrichment analysis done using transcripts generated from AB1-1 library as test set and AB2-1 as reference set with the FDR filter value 0.05.the 60 Go terms were differentially represented in these two libraries. Out of then 50 were over represented and 10 were under represented.

| **GO Term** | **Name** | **FDR** | **FWER** | **single test p-Value** | **# in test group** | **# in reference group** | **# non annot test** | **# non annot reference group** | **Over/**  **Under** |
| --- | --- | --- | --- | --- | --- | --- | --- | --- | --- |
| GO:0044444 | cytoplasmic part | 2.38E-04 | 5.95E-05 | 2.28E-06 | 194 | 164 | 178 | 291 | over |
| GO:0044424 | intracellular part | 0.010801 | 0.00824 | 2.06E-04 | 236 | 232 | 136 | 223 | over |
| GO:0043229 | intracellular organelle | 0.010801 | 0.010743 | 2.49E-04 | 183 | 168 | 189 | 287 | over |
| GO:0043226 | organelle | 0.010801 | 0.010743 | 2.49E-04 | 183 | 168 | 189 | 287 | over |
| GO:0032991 | macromolecular complex | 0.034219 | 0.041875 | 9.29E-04 | 59 | 39 | 313 | 416 | over |
| GO:0043231 | intracellular membrane-bounded organelle | 0.038615 | 0.06535 | 0.00142927 | 168 | 158 | 204 | 297 | over |
| GO:0043227 | membrane-bounded organelle | 0.038615 | 0.06535 | 0.00142927 | 168 | 158 | 204 | 297 | over |
| GO:0003676 | nucleic acid binding | 0.044051 | 0.093379 | 0.00198088 | 63 | 45 | 309 | 410 | over |
| GO:0009057 | macromolecule catabolic process | 0.044051 | 0.117417 | 0.00221608 | 21 | 8 | 351 | 447 | over |
| GO:0005488 | binding | 0.044051 | 0.120907 | 0.00238641 | 223 | 227 | 149 | 228 | over |
| GO:0003677 | DNA binding | 0.044051 | 0.122155 | 0.00245464 | 39 | 23 | 333 | 432 | over |
| GO:0005829 | cytosol | 0.044051 | 0.123816 | 0.0024708 | 18 | 6 | 354 | 449 | over |
| GO:0006508 | proteolysis | 0.047607 | 0.143379 | 0.00307076 | 23 | 10 | 349 | 445 | over |

Additional file 9; Table S2: Results of GO enrichment analysis done using transcripts generated from bulks of RILs as test set and SSH unigenes from individual parental libraries as reference set with the FDR filter value 0.05.the 13 Go terms were over represented in libraries from bulk of RILs.

| **GO Term** | **Name** | **FDR** | **FWER** | **single test p-Value** | **# in test group** | **# in reference group** | **# non annot test** | **# non annot reference group** | **Over/**  **Under** |
| --- | --- | --- | --- | --- | --- | --- | --- | --- | --- |
| GO:0006950 | response to stress | 1.92E-07 | 4.79E-08 | 1.69E-09 | 114 | 70 | 315 | 520 | over |
| GO:0009408 | response to heat | 8.75E-07 | 4.37E-07 | 1.74E-08 | 32 | 5 | 397 | 585 | over |
| GO:0009266 | response to temperature stimulus | 1.22E-06 | 9.15E-07 | 3.21E-08 | 44 | 13 | 385 | 577 | over |
| GO:0009628 | response to abiotic stimulus | 4.06E-05 | 4.06E-05 | 1.18E-06 | 69 | 39 | 360 | 551 | over |
| GO:0031224 | intrinsic to membrane | 4.83E-04 | 2.00E-04 | 7.54E-06 | 14 | 61 | 415 | 529 | under |
| GO:0044425 | membrane part | 4.83E-04 | 3.25E-04 | 1.12E-05 | 26 | 85 | 403 | 505 | under |
| GO:0016021 | integral to membrane | 4.83E-04 | 3.63E-04 | 1.31E-05 | 12 | 55 | 417 | 535 | under |
| GO:0050896 | response to stimulus | 0.00144219 | 0.00180112 | 4.44E-05 | 137 | 123 | 292 | 467 | over |
| GO:0009828 | plant-type cell wall loosening | 0.0127198 | 0.0220145 | 3.96E-04 | 9 | 0 | 420 | 590 | over |
| GO:0009827 | plant-type cell wall modification | 0.0127198 | 0.0220145 | 3.96E-04 | 9 | 0 | 420 | 590 | over |
| GO:0009642 | response to light intensity | 0.0134068 | 0.0297161 | 5.08E-04 | 11 | 1 | 418 | 589 | over |
| GO:0009644 | response to high light intensity | 0.0134068 | 0.0297161 | 5.08E-04 | 11 | 1 | 418 | 589 | over |
| GO:0010035 | response to inorganic substance | 0.0351312 | 0.0840903 | 0.00130965 | 37 | 23 | 392 | 567 | over |

Additional file 9; Table S3: Results of GO enrichment analysis done using transcripts generated from up regulated libraries (AS1-1 and AR1-1) as test set and unigenes from down regulatory libraries (AS2-1 and AR2-1) as reference set with the FDR filter value 0.05. The 10 Go terms were overrepresented in up regulated libraries and three GO terms were under represented.
