# Supplementary material for: Comparative analysis of expressed sequence tags (ESTs) between drought-tolerant and -susceptible genotypes of chickpea under terminal drought stress
Source: BMC Plant Biol. 2011 Apr 22;11:70. doi: 10.1186/1471-2229-11-70 (PMC3110109; doi:10.1186/1471-2229-11-70)
Supplement: Additional file 11 — Genotype specific response of chickpea unigenes in response to terminal drought stress. Expression profiling of differentially expressed ESTs generated by SSH libraries were analysed in drought stressed ICC 4958 and ICC 1882 using dot-blot expression analysis. Differential responses of unigenes are represented in normalised signal intensities values. Standard deviations are calculated from three different experiments. Signal intensity of Actin (GenBank: EU529707) used for normalisation of the signals between the blots and NPTII was used for signal background correction. Unigenes are listed according to their annotation generated in present work. [file 1471-2229-11-70-S11.DOCX]

| **S.No** | **EST Accession No.** | **Putative Function** | **BLASTX target species** | **BLASTX target species Accession No.** | **E-Value** | **ICC 4958** | **SD** | **ICC 1882** | **SD** | **ICC 4958/ICC 1882** |
| --- | --- | --- | --- | --- | --- | --- | --- | --- | --- | --- |
| 1 | HO062250 | calcium ion binding | Glycine max | ACU21229 | 1.68E-74 | 5.74 | 0.61 | 0.35 | 0.01 | 16.4 |
| 2 | HO062285 | Unknown | Glycine max | ACU23835 | 9.23E-24 | 1.07 | 0.04 | 0.07 | 0.03 | 15.3 |
| 3 | HO062911 | pathogenesis-related protein 10 | Glycine max | ACU16253 | 1.27E-06 | 5.17 | 0.12 | 0.56 | 0.04 | 9.2 |
| 4 | HO062281 | protein kinase | Ricinus communis | XP_002517344 | 1.35E-67 | 1.61 | 0.12 | 0.18 | 0.04 | 8.9 |
| 5 | HO062258 | protein | Populus trichocarpa | XP_002329781 | 1.77E-67 | 1.77 | 0.04 | 0.2 | 0.03 | 8.9 |
| 6 | HO062211 | ethylene overproducer-like 1 | S. lycopersicum | AAZ08351 | 8.08E-88 | 1.25 | 0.06 | 0.15 | 0.04 | 8.3 |
| 7 | HO062264 | cellular retinaldehyde-binding | Medicago truncatula | ABD28324 | 5.12E-71 | 0.9 | 0.06 | 0.21 | 0.01 | 4.3 |
| 8 | HO062310 | gdp-l-fucose synthase 1 | Glycine max | ACU23442 | 3.87E-38 | 6.95 | 0.24 | 1.85 | 0.04 | 3.8 |
| 9 | HO062435 | unknown [Glycine max] | Glycine max | ACU17911 | 5.82E-10 | 4.18 | 0.24 | 1.12 | 0.01 | 3.7 |
| 10 | HO062352 | protein | Vitis vinifera | CBI18920 | 4.03E-65 | 10.3 | 0.12 | 2.86 | 0.05 | 3.6 |
| 11 | HO062183 | cystathionine gamma-synthase | Medicago sativa | ABI34092 | 1.78E-108 | 0.61 | 0.02 | 0.17 | 0.01 | 3.6 |
| 12 | HO062422 | ribosomal protein l12 family protein | Glycine max | ACU14541 | 7.67E-51 | 6.24 | 0.26 | 1.78 | 0.1 | 3.5 |
| 13 | HO062275 | kinase associated protein phosphatase | Lotus japonicus | ACA57835 | 5.73E-112 | 10.2 | 0.02 | 3.1 | 0.14 | 3.3 |
| 14 | HO062705 | glutathione s-transferase | Medicago truncatula | ACJ84159 | 1.44E-29 | 1.95 | 0.09 | 0.61 | 0.02 | 3.2 |
| 15 | HO063146 | atp binding | Glycine max | ACU18280 | 8.54E-24 | 10.9 | 0.13 | 3.41 | 0.03 | 3.2 |
| 16 | HO062859 | protein | Populus trichocarpa | XP_002327102 | 3.92E-24 | 10.4 | 0.13 | 3.31 | 0.02 | 3.2 |
| 17 | HO062638 | nodulin 21 family protein | Glycine max | ACU19405 | 7.64E-77 | 9.53 | 0 | 3.05 | 0.14 | 3.1 |
| 18 | HO062698 | peroxidase | Glycine max | ACU24431 | 5.97E-47 | 7.39 | 0.25 | 2.37 | 0.03 | 3.1 |
| 19 | HO062737 | conserved hypothetical protein | Ricinus communis | XP_002527250 | 9.62E-12 | 9.48 | 0.07 | 3.35 | 0.04 | 2.8 |
| 20 | HO062198 | potassium transporter | Vitis vinifera | CAN69230 | 1.38E-52 | 1.92 | 0 | 0.68 | 0.03 | 2.8 |
| 21 | HO062721 | Unknown | NA |  |  | 0.47 | 0.05 | 0.17 | 0.06 | 2.8 |
| 22 | HO062417 | nhl repeat-containing protein | Vitis vinifera | XP_002265467 | 4.56E-92 | 0.44 | 0.01 | 0.16 | 0.03 | 2.8 |
| 23 | HO062226 | at1g78070 f28k19_28 | Ricinus communis | XP_002513893 | 3.39E-67 | 1.68 | 0.04 | 0.62 | 0.02 | 2.7 |
| 24 | HO062806 | nadp-dependent malic enzyme | Phaseolus vulgaris | CAA56354 | 5.40E-32 | 6.37 | 0.05 | 2.37 | 0.13 | 2.7 |
| 25 | HO062338 | Unknown | NA |  |  | 3.16 | 0.01 | 1.18 | 0.02 | 2.7 |
| 26 | HO062386 | esterase lipase thioesterase family protein | Arachis hypogaea | ACF74269 | 6.47E-38 | 9.88 | 0.76 | 3.75 | 0.61 | 2.6 |
| 27 | HO062232 | adenylate kinase | Medicago truncatula | ACJ86151 | 1.71E-89 | 0.81 | 0.02 | 0.31 | 0 | 2.6 |
| 28 | HO062690 | srg1-like protein | Glycine max | ACU24132 | 3.39E-38 | 0.13 | 0.03 | 0.05 | 0 | 2.6 |
| 29 | HO062328 | Unknown | NA |  |  | 11.3 | 0.42 | 4.38 | 0.32 | 2.6 |
| 30 | HO062244 | esterase lipase thioesterase family protein | Arabidopsis lyrata | XP_002891714 | 3.00E-80 | 8.88 | 0.66 | 3.45 | 0.91 | 2.6 |
| 31 | HO062736 | unknown [Medicago truncatula] | Medicago truncatula | ACJ84728 | 6.21E-05 | 11.3 | 0.06 | 4.39 | 0.23 | 2.6 |
| 32 | HO062733 | Unknown | NA |  |  | 5.59 | 0.58 | 2.2 | 0.07 | 2.5 |
| 33 | HO062976 | unknown [Glycine max] | Glycine max | ACU22842 | 1.68E-09 | 10.8 | 0.05 | 4.28 | 0.22 | 2.5 |
| 34 | HO062256 | Unknown | NA |  |  | 3.8 | 0.05 | 1.54 | 0.07 | 2.5 |
| 35 | HO062242 | fad nad binding | Glycine max | ACU14247 | 2.16E-41 | 0.65 | 0.08 | 0.27 | 0.06 | 2.4 |
| 36 | HO062295 | fmn binding protein | Glycine max | ACU22803 | 9.64E-43 | 8.93 | 0.05 | 3.73 | 0.03 | 2.4 |
| 37 | HO062267 | tfl1c protein | Glycine max | ABS57463 | 1.87E-24 | 0.67 | 0.09 | 0.28 | 0.04 | 2.4 |
| 38 | HO062272 | ac067971_24 ests gb | Vitis vinifera | CBI32019 | 1.45E-86 | 1.28 | 0.09 | 0.54 | 0.03 | 2.4 |
| 39 | HO062305 | cytochrome p450-like protein | Cicer arietinum | CAB56743 | 1.51E-101 | 3.8 | 0.02 | 1.61 | 0.01 | 2.4 |
| 40 | HO062273 | f-box and wd40 domain | Ricinus communis | XP_002517753 | 1.91E-46 | 2.31 | 0.11 | 0.98 | 0.06 | 2.4 |
| 41 | HO062555 | gdp dissociation inhibitor | Cicer arietinum | CAA06731 | 3.30E-111 | 10.2 | 0.09 | 4.36 | 0.18 | 2.3 |
| 42 | HO062180 | 1-aminocyclopropane-1-carboxylate synthase | Cicer arietinum | ABD16181 | 1.07E-59 | 7.95 | 0.05 | 3.44 | 0.11 | 2.3 |
| 43 | HO062366 | mads box | Pisum sativum | AAP83393 | 5.73E-42 | 6.89 | 0.3 | 3.01 | 0.16 | 2.3 |
| 44 | HO062461 | annexin p34 | Glycine max | ACU23990 | 1.56E-27 | 2.19 | 0.07 | 0.96 | 0.03 | 2.3 |
| 45 | HO062798 | proline-rich cell wall protein | Pisum sativum | CAA47812 | 2.26E-21 | 4.57 | 0.03 | 2.02 | 0.07 | 2.3 |
| 46 | HO062384 | flavonol synthase flavanone 3- | Glycine max | ACU23067 | 7.24E-102 | 6.68 | 0.03 | 3.07 | 0.05 | 2.2 |
| 47 | HO062660 | ubiquitin-associated ts-n domain-containing protein | Medicago truncatula | ACJ85071 | 1.26E-20 | 5.74 | 0.14 | 2.64 | 0.43 | 2.2 |
| 48 | HO062453 | transport protein | Populus trichocarpa | XP_002300208 | 1.05E-90 | 1.15 | 0.07 | 0.53 | 0.03 | 2.2 |
| 49 | HO062178 | mal d 1-associated protein | Glycine max | ACU15934 | 4.46E-18 | 9.25 | 0.96 | 4.27 | 0.89 | 2.2 |
| 50 | HO062414 | unknown [Glycine max] | Glycine max | ACU15064 | 4.83E-25 | 10.9 | 0.04 | 5.04 | 0.22 | 2.2 |
| 51 | HO062543 | nuclear transcription factor y subunit a- | Vitis vinifera | XP_002282043 | 2.86E-37 | 11.6 | 0.05 | 5.4 | 0.45 | 2.2 |
| 52 | HO062569 | chaperone binding | Vitis vinifera | CBI22961 | 1.28E-12 | 8.41 | 0.12 | 3.93 | 0.13 | 2.1 |
| 53 | HO062207 | esterase lipase thioesterase family protein | Ricinus communis | XP_002536932 | 4.00E-35 | 2.22 | 0.18 | 1.04 | 0.08 | 2.1 |
| 54 | HO062467 | nucleosome chromatin assembly factor group | Medicago truncatula | ACJ84798 | 1.72E-71 | 2.54 | 0.01 | 1.2 | 0 | 2.1 |
| 55 | HO062376 | carnitine acylcarnitine translocase | Ricinus communis | XP_002516060 | 5.72E-50 | 11.3 | 0.05 | 5.35 | 0.25 | 2.1 |
| 56 | HO062526 | arogenate dehydrogenase | Populus trichocarpa | XP_002331058 | 2.14E-77 | 1.64 | 0.12 | 0.78 | 0.02 | 2.1 |
| 57 | HO062452 | cytochrom p450-like protein | Glycine max | O48928 | 2.24E-89 | 1.28 | 0.06 | 0.61 | 0.04 | 2.1 |
| 58 | HO062448 | Unknown | NA |  |  | 4.32 | 0.03 | 2.06 | 0.03 | 2.1 |
| 59 | HO062633 | phospholipid cytidylyltransferase | Medicago truncatula | ACJ85759 | 3.27E-56 | 11.3 | 0.11 | 5.43 | 0.06 | 2.1 |
| 60 | HO062802 | ap2 erf domain-containing transcription factor | Populus trichocarpa | XP_002319909 | 7.80E-04 | 3.58 | 0.01 | 1.73 | 0.03 | 2.1 |
| 61 | HO062697 | Unknown | NA |  |  | 7.11 | 0.02 | 3.45 | 0.06 | 2.1 |
| 62 | HO062219 | udp-galactose transporter 3 | Glycine max | ACU17929 | 2.87E-63 | 2.1 | 0.02 | 1.02 | 0.03 | 2.1 |
| 63 | HO062797 | glyceraldehyde-3-phosphate dehydrogenase | Sorghum bicolor | XP_002452401 | 1.85E-36 | 5.39 | 0.04 | 2.62 | 0.05 | 2.1 |
| 64 | HO062308 | dna repair and transcription factor xpb1 | Arabidopsis lyrata | XP_002870639 | 4.69E-101 | 0.72 | 0.08 | 0.35 | 0.01 | 2.1 |
| 65 | HO062667 | 4-hydroxyphenylpyruvate dioxygenase | Arabidopsis lyrata | XP_002892349 | 1.91E-61 | 8.07 | 0 | 3.93 | 0.11 | 2.1 |
| 66 | HO062558 | unknown [Glycine max] | Glycine max | ACU23397 | 9.43E-05 | 11.2 | 0.2 | 5.48 | 0.05 | 2.0 |
| 67 | HO062433 | alkaline alpha galactosidase | Cicer arietinum | CAB71135 | 9.94E-117 | 3.88 | 0.76 | 1.92 | 0.13 | 2.0 |
| 68 | HO062354 | beta-galactosidase-complementation protein | Triticum aestivum | CAQ43070 | 7.68E-37 | 10.7 | 0.06 | 5.33 | 0.28 | 2.0 |
| 69 | HO062227 | dual specificity protein | Vitis vinifera | XP_002274406 | 1.22E-94 | 1.66 | 0.11 | 0.83 | 0.01 | 2.0 |
| 70 | HO062833 | luminal binding protein | Populus trichocarpa | XP_002303672 | 7.11E-18 | 8.6 | 0.06 | 4.45 | 0.1 | 1.9 |
| 71 | HO062402 | 23kda polypeptide of the oxygen evolving complex of photosystem ii | Glycine max | ACU23674 | 1.67E-64 | 2.46 | 0.07 | 1.28 | 0.02 | 1.9 |
| 72 | HO062184 | guanosine-3 -bis 3 - | Medicago truncatula | CAJ00006 | 1.91E-104 | 2.08 | 0.12 | 1.09 | 0.01 | 1.9 |
| 73 | HO062856 | unknown [Glycine max] | Glycine max | ACU21365 | 4.11E-27 | 6.2 | 0.07 | 3.27 | 0.22 | 1.9 |
| 74 | HO062574 | protein | Populus trichocarpa | XP_002329084 | 7.95E-102 | 4.07 | 0.05 | 2.16 | 0.04 | 1.9 |
| 75 | HO062549 | predicted protein [Populus trichocarpa] | Populus trichocarpa | XP_002315580 | 7.99E-06 | 11.4 | 0.07 | 6.07 | 0.01 | 1.9 |
| 76 | HO062800 | Unknown | NA |  |  | 6.06 | 0.05 | 3.24 | 0.06 | 1.9 |
| 77 | HO062474 | leucine-rich repeat protein | Medicago truncatula | ACJ86048 | 7.87E-89 | 2.47 | 0.23 | 1.33 | 0.08 | 1.9 |
| 78 | HO062431 | glutathione transferase 10 | Glycine max | ACU20910 | 1.37E-35 | 3.99 | 0.03 | 2.16 | 0.04 | 1.8 |
| 79 | HO062268 | Unknown | NA |  |  | 8.15 | 0.07 | 4.43 | 0.11 | 1.8 |
| 80 | HO062776 | 60s ribosomal protein l10a-1 | Glycine max | ACU13284 | 1.37E-27 | 4.74 | 0.06 | 2.58 | 0.07 | 1.8 |
| 81 | HO062460 | protein | Arabidopsis lyrata | XP_002888634 | 2.24E-14 | 5.05 | 0.07 | 2.76 | 0 | 1.8 |
| 82 | HO062379 | betaine aldehyde dehydrogenase | Pisum sativum | 3IWK_A | 8.45E-111 | 0.78 | 0.02 | 0.43 | 0.03 | 1.8 |
| 83 | HO063154 | f-box and wd40 domain | Vitis vinifera | XP_002267260 | 6.05E-12 | 11.9 | 0.08 | 6.57 | 0.02 | 1.8 |
| 84 | HO062795 | retrotransposon protein | Persephonella marina | YP_002730924 | 1.46E-04 | 9.12 | 0.05 | 5.06 | 0.06 | 1.8 |
| 85 | HO062939 | class 10 pr protein | Medicago sativa | CAC37691 | 1.67E-32 | 10.8 | 0.01 | 6.02 | 0.09 | 1.8 |
| 86 | HO062249 | cinnamoyl reductase | Glycine max | ACU20060 | 2.99E-59 | 2.88 | 0.02 | 1.61 | 0.02 | 1.8 |
| 87 | HO062896 | harpin-induced hin1-related harpin-responsive | Populus trichocarpa | XP_002318799 | 3.91E-09 | 4.88 | 0.07 | 2.74 | 0.05 | 1.8 |
| 88 | HO062839 | Unknown | NA |  |  | 7.15 | 0.04 | 4.04 | 0.05 | 1.8 |
| 89 | HO062693 | Unknown | NA |  |  | 0.76 | 0.09 | 0.43 | 0.03 | 1.8 |
| 90 | HO062175 | myosin heavy chain-related | Vitis vinifera | CBI22439 | 9.58E-13 | 3.23 | 0.1 | 1.84 | 0.04 | 1.8 |
| 91 | HO062783 | tonoplast intrinsic protein | Cicer arietinum | CAD33928 | 6.26E-23 | 1.11 | 0.02 | 0.64 | 0.03 | 1.7 |
| 92 | HO062217 | protein kinase | Medicago truncatula | ABN08751 | 1.95E-65 | 1.35 | 0.06 | 0.78 | 0.02 | 1.7 |
| 93 | HO062805 | unknown [Glycine max] | Glycine max | ACU16843 | 4.43E-04 | 5.64 | 0.13 | 3.27 | 0.05 | 1.7 |
| 94 | HO062796 | protein | Ricinus communis | XP_002510282 | 8.06E-10 | 2.48 | 0.02 | 1.44 | 0.02 | 1.7 |
| 95 | HO062940 | isoflavone synthase 2 | Cicer arietinum | CAB50768 | 1.11E-24 | 7.24 | 0.01 | 4.21 | 0.02 | 1.7 |
| 96 | HO062392 | transcription regulator, putative | Ricinus communis | XP_002518574 | 8.37E-12 | 3.09 | 0.03 | 1.81 | 0.12 | 1.7 |
| 97 | HO063143 | saur family protein | Glycine max | ACU16794 | 7.60E-53 | 12.2 | 0.29 | 7.15 | 0.02 | 1.7 |
| 98 | HO062823 | protein | Astragalus sinicus | ADK35105 | 3.36E-26 | 7.28 | 0.02 | 4.29 | 0.05 | 1.7 |
| 99 | HO062716 | Unknown | NA |  |  | 2.04 | 0.02 | 1.21 | 0.01 | 1.7 |
| 100 | HO062877 | actin depolymerizing factor 6 | Glycine max | ACU19431 | 1.30E-23 | 6.08 | 0.04 | 3.61 | 0.01 | 1.7 |
| 101 | HO063136 | s-adenosylmethionine synthetase | Medicago truncatula | A4PU48 | 4.68E-89 | 5.69 | 0.06 | 3.38 | 0.13 | 1.7 |
| 102 | HO062365 | glycosyl hydrolase family 18 protein | Medicago truncatula | ACJ86301 | 5.83E-46 | 7.08 | 0.04 | 4.23 | 0.02 | 1.7 |
| 103 | HO062874 | Unknown | NA |  |  | 3.52 | 0.22 | 2.12 | 0.03 | 1.7 |
| 104 | HO062718 | cysteine protease | Brassica napus | AAB53103 | 1.06E-17 | 11.3 | 0.09 | 6.81 | 0.03 | 1.7 |
| 105 | HO062394 | lipid transfer protein | Tamarix hispida | ACM78620 | 1.50E-13 | 4.43 | 0.02 | 2.72 | 0.07 | 1.6 |
| 106 | HO064407 | type 2a phosphatase activator | Glycine max | ACU24279 | 2.99E-22 | 2.69 | 0.08 | 1.67 | 0.02 | 1.6 |
| 107 | HO062197 | Unknown | NA |  |  | 4.89 | 0.09 | 3.04 | 0.03 | 1.6 |
| 108 | HO062520 | protein | Ricinus communis | XP_002533392 | 1.53E-34 | 4.15 | 0.05 | 2.6 | 0.04 | 1.6 |
| 109 | HO062284 | transcription elongation factor | Vitis vinifera | XP_002281873 | 7.56E-67 | 2.33 | 0.08 | 1.46 | 0.06 | 1.6 |
| 110 | HO063186 | arabinogalactan protein | Glycine max | ACU18441 | 2.97E-33 | 11.6 | 0.06 | 7.29 | 0.01 | 1.6 |
| 111 | HO062818 | Unknown | NA |  |  | 10.7 | 0.06 | 6.72 | 0.02 | 1.6 |
| 112 | HO062406 | Unknown | NA |  |  | 4.19 | 0.07 | 2.63 | 0.03 | 1.6 |
| 113 | HO062868 | Unknown | NA |  |  | 4.1 | 0.04 | 2.58 | 0.02 | 1.6 |
| 114 | HO062846 | ubiquitin | Solanum lycopersicum | CAA51679 | 5.27E-51 | 6.19 | 0.08 | 3.92 | 0.02 | 1.6 |
| 115 | HO062572 | protein | Ricinus communis | XP_002514138 | 9.85E-43 | 8.83 | 0.14 | 5.61 | 0.04 | 1.6 |
| 116 | HO063135 | dna-binding protein | Medicago truncatula | ACK38184 | 7.78E-09 | 11.1 | 0.03 | 7.05 | 0.06 | 1.6 |
| 117 | HO062866 | cytosolic class i small heat shock protein 3b | Glycine max | P05478 | 2.81E-37 | 4.82 | 0.05 | 3.07 | 0.03 | 1.6 |
| 118 | HO062793 | Unknown | NA |  |  | 7.43 | 0.09 | 4.75 | 0.05 | 1.6 |
| 119 | HO062855 | protein | Populus trichocarpa | XP_002328172 | 7.44E-35 | 3.7 | 0.07 | 2.37 | 0.04 | 1.6 |
| 120 | HO062176 | at5g17920 mpi7_60 | Cicer arietinum | ACL14488 | 8.74E-24 | 6.42 | 0.06 | 4.12 | 0.01 | 1.6 |
| 121 | HO062363 | myb-related transcription factor | Glycine max | ACU24041 | 1.33E-26 | 3.41 | 0.07 | 2.19 | 0.04 | 1.6 |
| 122 | HO062502 | quinone oxidoreductase | Medicago truncatula | ACJ84736 | 1.75E-90 | 1.91 | 0.07 | 1.23 | 0.04 | 1.6 |
| 123 | HO062598 | Unknown | NA |  |  | 9.53 | 0.12 | 6.14 | 0.02 | 1.6 |
| 124 | HO062399 | metal ion binding | Vitis vinifera | XP_002284075 | 6.75E-31 | 5.83 | 0.06 | 3.76 | 0.07 | 1.6 |
| 125 | HO062506 | ribosomal protein s6 | Glycine max | ACU18357 | 2.64E-18 | 5.02 | 0.06 | 3.24 | 0.05 | 1.5 |
| 126 | HO062790 | chalcone synthase | Trifolium subterraneum | P51083 | 1.43E-11 | 7.97 | 0.03 | 5.17 | 0.06 | 1.5 |
| 127 | HO062843 | phosphatase subunit g4-1 | Ricinus communis | XP_002516887 | 1.28E-44 | 3.85 | 0.06 | 2.5 | 0 | 1.5 |
| 128 | HO062811 | benzoquinone reductase | Glycine max | ACU19740 | 2.21E-56 | 3.73 | 0.07 | 2.43 | 0.07 | 1.5 |
| 129 | HO062840 | cytochrome p450 monooxygenase cyp83e8 | Cicer arietinum | CAD31843 | 3.98E-19 | 6.04 | 0.07 | 3.94 | 0.03 | 1.5 |
| 130 | HO062192 | Unknown | NA |  |  | 3.98 | 0.01 | 2.6 | 0.04 | 1.5 |
| 131 | HO062885 | at5g26830 f2p16_90 | Vitis vinifera | XP_002265302 | 4.28E-44 | 3.61 | 0.01 | 2.37 | 0.16 | 1.5 |
| 132 | HO062867 | Unknown | NA |  |  | 10.9 | 0.04 | 7.18 | 0.02 | 1.5 |
| 133 | HO062723 | pollen ole e 1 allergen and extensin family protein | Ricinus communis | XP_002522975 | 9.60E-43 | 0.92 | 0.06 | 0.61 | 0.14 | 1.5 |
| 134 | HO063202 | sulfate transporter | Vitis vinifera | XP_002285810 | 4.52E-112 | 2.32 | 0.08 | 1.54 | 0.02 | 1.5 |
| 135 | HO062706 | Unknown | NA |  |  | 3.81 | 0.05 | 2.53 | 0.03 | 1.5 |
| 136 | HO062529 | protein | Vitis vinifera | XP_002264608 | 2.23E-18 | 0.75 | 0.05 | 0.5 | 0.04 | 1.5 |
|  |  |  |  |  |  |  |  |  |  |  |
| 1 | HO062991 | Unknown | NA |  |  | 0.05 | 0.01 | 7.48 | 0.33 | 149.6 |
| 2 | HO063105 | Unknown | NA |  |  | 2.27 | 0.15 | 9.52 | 0.11 | 4.194 |
| 3 | HO063060 | Unknown | NA |  |  | 3.25 | 0.13 | 10.5 | 0.23 | 3.231 |
| 4 | HO063129 | fructose-bisphosphate aldolase | Cicer arietinum | O65735 | 1.94E-13 | 3.36 | 0.29 | 10.6 | 0.11 | 3.143 |
| 5 | HO063040 | protein | Oryza sativa | NP_001053957 | 1.11E-53 | 3.17 | 0.09 | 9.55 | 0.22 | 3.013 |
| 6 | HO063086 | 60s ribosomal protein l23 | Castanea sativa | AAK25758 | 2.16E-34 | 3.61 | 0.3 | 10.7 | 0.29 | 2.956 |
| 7 | HO063090 | ribosomal protein l3 | Selaginella moellendorffii | XP_002968198 | 1.04E-46 | 3.48 | 0.45 | 7.75 | 0.25 | 2.227 |
| 8 | HO063042 | metal ion binding | Ricinus communis | XP_002510790 | 1.49E-05 | 5.42 | 0.37 | 11.7 | 0.32 | 2.151 |
| 9 | HO063043 | Unknown | NA |  |  | 3.21 | 0.1 | 6.79 | 0.18 | 2.115 |
| 10 | HO063022 | chalcone synthase | Medicago sativa | P51078 | 3.02E-33 | 5.14 | 0.08 | 10.8 | 0.06 | 2.109 |
| 11 | HO063107 | polyubiquitin | Cucumis sativus | ACZ69386 | 7.27E-45 | 4.08 | 0.14 | 8.34 | 0.38 | 2.044 |
| 12 | HO063079 | stress induced protein | Glycine max | ACU24578 | 2.13E-12 | 3.77 | 0.22 | 7.69 | 0.28 | 2.04 |
| 13 | HO063041 | heavy-metal-associated domain-containing expressed | Ricinus communis | XP_002510790 | 1.69E-06 | 4.26 | 0.14 | 8.66 | 0.23 | 2.033 |
| 14 | HO063089 | Unknown | NA |  |  | 3.28 | 0.11 | 6.66 | 0.16 | 2.03 |
| 15 | HO063082 | polyubiquitin | Vitis vinifera | CBI22354 | 1.86E-119 | 4.82 | 0.21 | 9.66 | 0.12 | 2.004 |
| 16 | HO063124 | pathogenesis related protein | Cicer arietinum | CAA56142 | 5.57E-82 | 3.54 | 0.31 | 7.08 | 0.16 | 2 |
| 17 | HO063093 | Unknown | NA |  |  | 3.86 | 0.08 | 7.42 | 0.15 | 1.922 |

**Additional File 11: Genotype specific response of chickpea unigenes in response to terminal drought stress.**

Expression profiling of differentially expressed ESTs generated by SSH libraries were analysed in drought stressed ICC 4958 and ICC 1882 using dot-blot expression analysis. Differential responses of unigenes are represented in normalised signal intensities values. Standard deviations are calculated from three different experiments. Signal intensity of Actin (GenBank: EU529707) used for normalisation of the signals between the blots and NPTII was used for signal background correction. Unigenes are listed according to their annotation generated in present work.
